# Global ubiquitination analysis reveals extensive modification and proteasomal degradation of cowpox virus proteins, but preservation of viral cores

**DOI:** 10.1038/s41598-018-20130-9

**Published:** 2018-01-29

**Authors:** Marica Grossegesse, Joerg Doellinger, Annemarie Fritsch, Michael Laue, Janett Piesker, Lars Schaade, Andreas Nitsche

**Affiliations:** 1 Robert Koch Institute, Centre for Biological Threats and Special Pathogens: Highly Pathogenic Viruses (ZBS 1), Berlin, 13353 Germany; 2 Robert Koch Institute, Centre for Biological Threats and Special Pathogens: Proteomics and Spectroscopy (ZBS 6), Berlin, 13353 Germany; 3 Robert Koch Institute, Centre for Biological Threats and Special Pathogens: Advanced Light and Electron Microscopy (ZBS 4), Berlin, 13353 Germany; 4 Robert Koch Institute, Centre for Biological Threats and Special Pathogens, Berlin, 13353 Germany

## Abstract

The emergence of Variola virus-like viruses by natural evolution of zoonotic Orthopoxviruses, like Cowpox virus (CPXV), is a global health threat. The proteasome is essential for poxvirus replication, making the viral components interacting with the ubiquitin-proteasome system attractive antiviral targets. We show that proteasome inhibition impairs CPXV replication by prevention of uncoating, suggesting that uncoating is mediated by proteasomal degradation of viral core proteins. Although Orthopoxvirus particles contain considerable amounts of ubiquitin, distinct modification sites are largely unknown. Therefore, for the first time, we analyzed globally ubiquitination sites in CPXV mature virion proteins using LC-MS/MS. Identification of 137 conserved sites in 54 viral proteins among five CPXV strains revealed extensive ubiquitination of structural core proteins. Moreover, since virions contained primarily K48-linked polyubiquitin, we hypothesized that core proteins are modified accordingly. However, quantitative analysis of ubiquitinated CPXV proteins early in infection showed no proteasomal degradation of core proteins. Instead, our data indicate that the recently suggested proteasomal regulation of the uncoating factor E5 is a prerequisite for uncoating. Expanding our understanding of poxvirus uncoating and elucidating a multitude of novel ubiquitination sites in poxvirus proteins, the present study verifies the major biological significance of ubiquitin in poxvirus infection.

## Introduction

The ubiquitin-proteasome system (UPS) is exploited by members of most virus families^1^ and is essential for the replication of different virus families, e.g. *Pox*-^2–4^, *Reo*-^5^ and *Coronaviridae*^6^. As a member of the *Poxviridae* family the genus *Orthopoxvirus* (OPV) comprises complex enveloped DNA viruses, including the smallpox-causing Variola virus (VARV), Vaccinia virus (VACV), which was used for smallpox eradication^7^, and several animal-borne viruses with zoonotic potential like Cowpox virus (CPXV)^8–10^. Having caused the death of hundreds of millions of people, VARV is the most prominent member of OPV. Although VARV has been eradicated in 1979, it is still a global health threat because of its possible release in the context of a bioterrorist attack and the emergence of VARV-like viruses from circulating OPV by natural evolution^11^. CPXV are well suited to fill the niche that was created by VARV eradication, since it has the largest OPV genome including homologous open reading frames of all VARV genes. Currently, the number of zoonotic infections in Europe caused by CPXV is increasing, illustrating the need for a comprehensive understanding of the virus biology, especially the interaction with the host cell^12,13^.

The ubiquitin-proteasome pathway is highly modulated during OPV infection, e.g. by virus-encoded ubiquitin ligases or viral BTB/kelch and ANK/PRANC proteins that interact with cellular ubiquitin ligases^14,15^. OPV do not encode viral ubiquitin, but host ubiquitin locates to viral replication sites during infection^16^. Additionally, several proteome studies identified ubiquitin as part of the VACV and CPXV mature virion^17–19^ and it was found to be more than 100-fold enriched in OPV particles compared to human cells^17^. Although ubiquitin is obviously an important poxvirus protein modification, only sparse information is available about ubiquitinated virus proteins.

Infectious OPV particles consist of a DNA-containing core which is surrounded by one or two membranes, resulting in intracellular mature virions (IMV) or extracellular enveloped virions (EEV), respectively. Two lateral bodies (LBs) are located between core and membrane, which are assumed to deliver viral enzymes into the host cell cytoplasm early in infection^20^. Poxvirus replication exclusively takes place within the cytoplasm of the host cell. Upon entry, the intact core is delivered into the cytoplasm, followed by early viral gene expression inside the core. After the viral DNA is released by core uncoating, replication as well as intermediate, and late viral gene expression proceed in delimited cytoplasmic areas called virus factories (VF)^21,22^.

The role of the UPS in OPV infection has been mostly analyzed using VACV, which is the best characterized OPV. Treatment of HeLa cells with proteasome inhibitors led to reduced viral titers and impaired intermediate and late viral gene expression, while early viral gene expression still occurred. Also, the prevention of VACV-DNA replication and VF formation was observed in the context of proteasome inhibition^2–4^. This is consistent with the finding that proteasome activity is required for VACV genome uncoating^4^.

In contrast to VACV, the role of the UPS during CPXV infection has been barely investigated so far. Treatment of CPXV-infected cells with proteasome inhibitors prevents late protein expression and reduces viral titers^3^ but it remains unknown to what extent results generated with VACV apply to CPXV. Hence, we aimed to characterize the role of ubiquitin and the UPS in CPXV infection in a more comprehensive way. We initially verified the inhibition of proteasome activity by MG-132 and Bortezomib in HeLa cells and excluded cytotoxic effects. Subsequently, we showed that proteasome inhibition impairs CPXV replication by prevention of viral core degradation, resulting in early but not in late viral protein expression. Therefore, we hypothesized that proteasomal degradation of K48-linked polyubiquitinated core proteins is the mechanism underlying CPXV uncoating, as previously suggested for VACV^4^. Since this hypothesis has not been verified yet, we aimed to prove it by conducting the first global LC-MS/MS-based ubiquitinome analysis of OPV IMV proteins. Using purified CPXV IMV particles, we identified 137 conserved ubiquitination sites in 54 viral proteins among five CPXV strains. As hypothesized, CPXV major core proteins were extensively ubiquitinated. But surprisingly, we were unable to detect their proteasome-dependent degradation upon infection. Instead, 54 other viral proteins were degraded in a proteasome-dependent manner early in infection, including all proteins associated with the formation of viral pre-replication sites^23^. Most notably, we detected the proteasomal degradation of the uncoating factor E5 (VACV D5), which is likely a prerequisite for OPV genome uncoating^23^.

Our data deepen the understanding of OPV genome uncoating and provide evidence of extensive ubiquitin modification in OPV proteins. Moreover, the multitude of novel modification sites identified in OPV proteins of diverse functions underlines that the biological significance of ubiquitination for OPV is far from being restricted to uncoating.

## Results and Discussion

### MG-132 and Bortezomib inhibit proteasome activity without inducing cytotoxic effects

**Figure 1:**
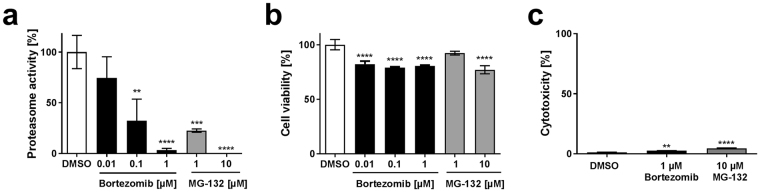
Effect of proteasome inhibitors on HeLa cells. HeLa cells were incubated for 24 h in the presence of MG-132, Bortezomib or DMSO and assayed for (**a**) chymotrypsin-like proteasome activity, (**b**) ATP amount and (**c**) release of lactate dehydrogenase. Error bars indicate means ± standard deviation of one experiment performed at least in triplicate. Statistics: One-way ANOVA and Bonferroni’s multiple comparisons test (*p ≤ 0.05, **p ≤ 0.01, ***p ≤ 0.001, ****p ≤ 0.0001).

In order to analyze the effect of proteasome inhibition on CPXV replication, the proteasome activity in HeLa cells treated with two different inhibitors was tested. For this purpose, different concentrations of MG-132 and Bortezomib were analyzed for their inhibition of the chymotryptic-like proteasome activity in HeLa cells. Both compounds inhibit primarily the chymotrypsin-like site of the 26 S proteasome but may also act on the caspase-like site in a concentration-dependent manner^24^. A complete inhibition of proteasome activity was only observed using 10 µM MG-132 and 1 µM Bortezomib, which represented the highest inhibitor concentrations tested (Fig. 1a). These concentrations have already been applied in previous studies, though proteasome inhibition has not been shown^2,3^. Analysis of the ATP amount revealed about 80% cell viability of inhibitor-treated cells after 24 h of treatment (Fig. 1b). Inhibitor-induced release of lactate dehydrogenase, as a measure of cytotoxicity, was below 5% (Fig. 1c), confirming acceptable conditions for infection experiments.

### Proteasome inhibition impairs CPXV replication, virus factory formation and late protein synthesis

**Figure 2:**
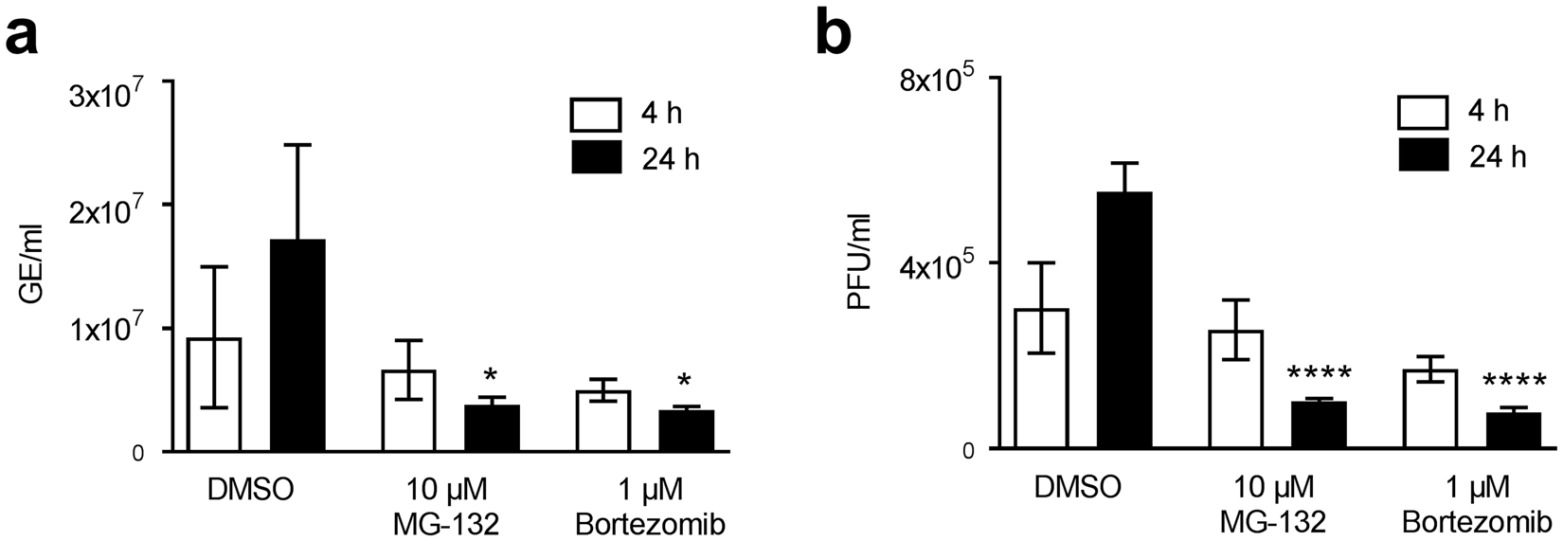
Proteasome inhibition leads to reduced CPXV genome equivalents and infectious particles. HeLa cells were infected with CPXV BR at an MOI of 1 in the presence or absence of 10 µM MG-132 or 1 µM Bortezomib. At 4 and 24 h post infection the amount of (**a**) viral genome equivalents (GE) and (**b**) plaque-forming units (PFU) in the supernatant was analyzed. Error bars indicate means ± standard deviation of one experiment performed in triplicate. Statistics: One-way ANOVA and Bonferroni’s multiple comparisons test (*p ≤ 0.05, ****p ≤ 0.0001).

**Figure 3:**
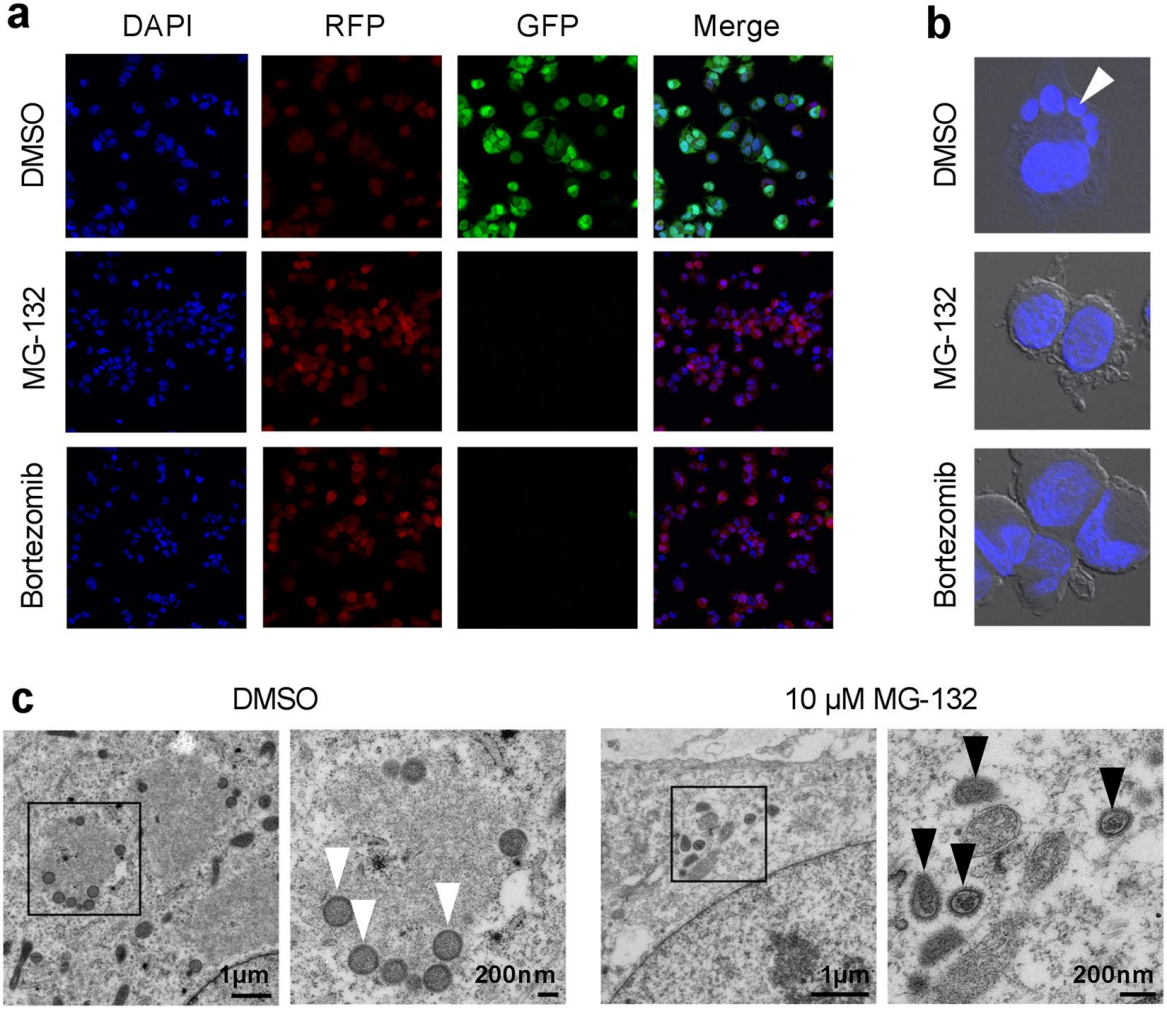
Proteasome inhibition impairs CPXV late protein expression and virus factory formation by prevention of uncoating. (**a**,**b**) HeLa cells were infected with CPXV BRFseR expressing RFP under an early viral promotor and GFP under a late viral promotor at an MOI of 5 in the presence or absence of 10 µM MG-132 or 1 µM Bortezomib. After 8 h cells were fixed and stained with DAPI. (**a**) Inhibitor-treated cells display no late protein expression, while early protein expression is still detectable. RFP fluorescence in control cells is overlaid by GFP fluorescence and hence appears less intense. (**b**) While cells with non-inhibited proteasome (DMSO) display typical virus factories in the cytoplasm (white triangle), these structures were absent in proteasome inhibitor-treated cells. (**c**) HeLa cells were infected with CPXV BRFseR at an MOI of 100 in the presence of 10 µM MG-132 or DMSO and subjected to transmission electron microscopy 4 h post infection. Virus factories and immature virus particles (white arrows) were only detected in cells treated with DMSO, while intact cores (black arrows) were regularly found in cells treated with proteasome inhibitor MG-132.

Since reduction of CPXV infectious particles has only been described for proteasome inhibition using Bortezomib^3^, we tested the effect of MG-132 which is the inhibitor primarily used for studies with VACV^2–4^. We were able to confirm a reduction of CPXV genome copies and plaque-forming units (PFU) in HeLa cells treated with either Bortezomib or MG-132 (Fig. 2). Inhibition of late protein expression has only been shown using MG-132^3^, while inhibition of VF formation has not been shown for CPXV at all. HeLa cells infected with a recombinant CPXV strain expressing differently fluorescent proteins under control of an early and a late viral promotor revealed the absence of late protein expression in inhibitor-treated cells while early protein expression was still detectable (Fig. 3a). Additionally, VF were only detected in control cells with non-inhibited proteasome, while VF were absent in cells with inhibited proteasome (Fig. 3b). Taken together, we demonstrated that the effects of proteasome inhibition observed for VACV replication are transferable to CPXV.

### Proteasome inhibition leads to intact virus cores in the cytoplasm

The absence of late protein expression and viral genome replication has been hypothesized to result from the inability of core uncoating. Therefore, we wondered whether we could detect intact CPXV cores in the cytoplasm of cells with inhibited proteasome as demonstrated for VACV^2,4^. Using electron microscopy, we were indeed able to detect intact CPXV cores in 72% of MG-132-treated cells, while only 1% of cells with non-inhibited proteasome showed intact core structures. Furthermore, VF were found in 77% of cells with non-inhibited proteasome, while no VF were detected in MG-132-treated cells (Fig. 3c). Both results suggest that CPXV virus replication is impaired by MG-132 treatment due to prevention of core uncoating.

Most previous data^2,4,25^, including our own, indicated that proteasome-dependent degradation of major core proteins is the mechanism underlying OPV genome uncoating. Using a confocal microscopy-based core stabilization assay, Satheshkumar and colleagues still observed 40–45% of uncoated cores in cells treated with MG-132. Although they additionally observed a more than 2-fold increase in intact virus cores in the presence of MG-132, they concluded that the inability of uncoating is not sufficient to account for the complete replication inhibition of VACV observed in proteasome-inhibited cells^2^. However, virus attachment to cells at 4 °C for 1 h without the presence of MG-132 prior to 37 °C incubation in the presence of proteasome inhibitor, as done by Satheshkumar and colleagues^2^, may result in uncoating even before complete proteasome inhibition is achieved. This may explain the observed 40–45% of uncoated cores in the presence of MG-132^2^. We still observed residual proteasome activity after 1 h of MG-132 treatment (data not shown) verifying this assumption and hence we pretreated cells for electron microscopy with proteasome inhibitor prior to infection. Confirming that proteasome inhibition prevents CPXV core degradation, we aimed to identify ubiquitination sites in structural core proteins, which are a prerequisite for their proteasome-dependent degradation. Therefore, we conducted the first global LC-MS/MS-based ubiquitinome analysis of virion proteins.

### Analysis of the CPXV mature virion ubiquitinome

Although K48-linked polyubiquitin was identified in VACV core fractions using immunoblotting^4^, no information is available about distinct ubiquitinated proteins in the core fraction that would prove the hypothesis of ubiquitinated structural core proteins. This encouraged us to analyze the mature virion ubiquitinome of five CPXV strains to elucidate the conserved CPXV ubiquitinome. Besides the cell culture-adapted CPXV strain Brighton Red (BR) representing the best-characterized CPXV strain, we analyzed four CPXV strains that were isolated between 2007 and 2009 in the German Consultant Laboratory for Poxviruses at the Robert Koch Institute, Germany. All of these four strains led to documented zoonotic infections. If the hypothesis of ubiquitinated core proteins is true, ubiquitination sites in corresponding proteins should be identifiable. Therefore, we purified CPXV mature virions by density gradient ultracentrifugation, lysed the purified particles and digested the proteins with trypsin. Ubiquitinated lysine residues resulted in diGly(K) remnants at peptides, which were subsequently enriched via immunoaffinity purification and analyzed by LC-MS/MS.

In total, between 656 and 1,230 diGly(K) peptides were identified from about 12,000 to 14,500 fragment spectra, depending on the CPXV strain (Supplementary Table S1). About 50% of these peptides (based on peptide area quantification) contained at least one ubiquitination site, indicating a successful enrichment of diGly(K) peptides. As expected, the proportion of missed cleavages was greatly increased for peptides with diGly(K) residue since trypsin cleavage is prevented by diGly(K) remnants^26^. Furthermore, it was not surprising that about 58% of ubiquitinated peptides (based on peptide area quantification) originated from ubiquitin itself. Viral peptides made up 34% of ubiquitinated peptides while only 8% originated from the host or contaminants (Supplementary Table S2). Hence, ubiquitin is probably derived from viral rather than host proteins. The ubiquitin-like proteins ISG15 and NEDD8 also result in diGly(K) remnants after trypsin digestion^26^, but since these peptides together made up less than 0.2% of all peptide areas, diGly(K) residues were considered to be ubiquitin specific.

**Figure 4:**
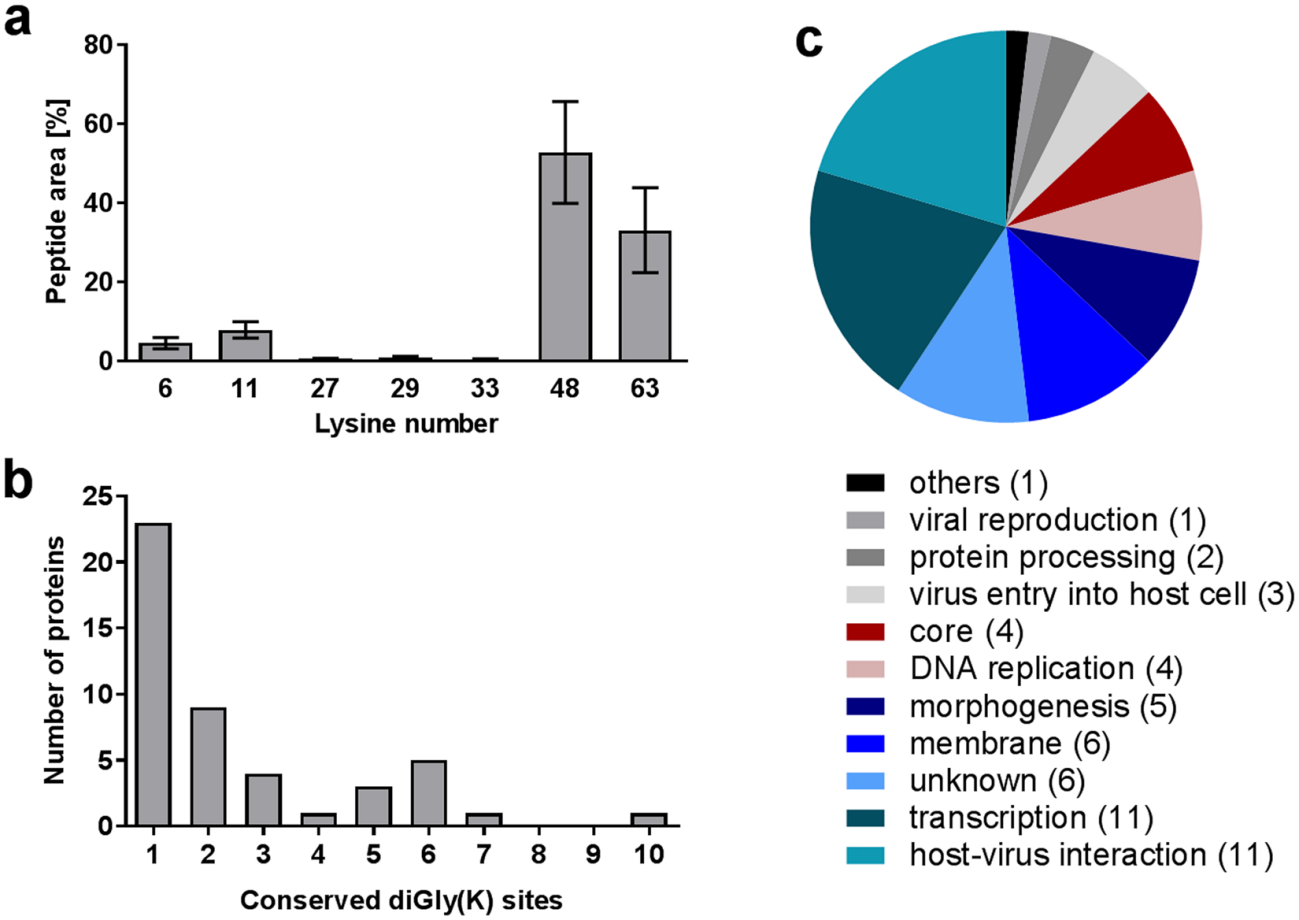
Conserved ubiquitination sites and lysine linkages in CPXV mature virions. (**a**) Quantitative approximation of lysine linkages in CPXV IMV particles using linkage-specific peptide areas. Shown is the mean ± standard deviation of five CPXV strains in % of total ubiquitin diGly(K) peptide area. (**b**) Number of conserved ubiquitination sites in CPXV proteins. (**c**) CPXV proteins with conserved ubiquitination sites grouped by function. Shown is the number of proteins (in parenthesis) associated with a distinct function.

Ubiquitin itself contains seven lysine residues at positions 6, 11, 27, 29, 33, 48 and 63 by which polyubiquitin chains can be built, leading to different downstream reactions^27^. While K48-linked polyubiquitin chains are established to mediate proteasomal degradation, K63 linkages are associated with non-proteolytic functions^27,28^. Also, K11-linked polyubiquitin can lead to proteasomal degradation, while less is known about the fate of proteins tagged with K6, K27, K29 and K33 polyubiquitin chains^27^. All of the seven ubiquitin linkages possible were identified in all five CPXV strains, except for K33 which was not identified in the CPXV RatHei sample. Quantitative approximation of lysine linkages using peptide areas of linkage-specific ubiquitin peptides revealed K48-linked polyubiquitin as the most abundant ubiquitin linkage in CPXV IMV particles, with a mean of 53% of all ubiquitin linkages. K63 was identified to be the second-most abundant linkage, with a mean portion of 33% in CPXV mature virions. K11 and K6 represented about 8% and 4.5%, respectively, while K27 and K29 linkages made up less than 1% each (Fig. 4a and Supplementary Table S3). These abundances resemble the ubiquitin linkage frequencies observed in cells^29^. Not surprisingly, it seems that the CPXV ubiquitin linkage abundance is well adapted to its host.

K48 is probably the predominant ubiquitin linkage in CPXV IMV proteins, indicating that at least some virion proteins are tagged for proteasomal degradation. This supports the hypothesis of K48-linked polyubiquitinated core proteins. It should be noted that peptide area quantification of ubiquitin linkages relies on the comparison of non-identical peptides and hence represents only a rough approximation. Moreover, from our results, it is not possibly to determine the precise ubiquitination linkage of distinct virion proteins. However, in agreement with our results, Mercer and colleagues identified K48-linked polyubiquitin in VACV virion core fractions by Western blotting but, in contrast to our results, they were not able to detect K63-linked polyubiquitin chains^4^. This might be attributed to the lesser abundance of K63 linkages which may not be detectable using Western blot but it may also represent a biological difference between VACV and CPXV. It should be noted further that fractionation of purified IMV particles in core and membrane fraction by incubation with detergent and reduction agents^4,20,30^ as performed by Mercer and colleagues^4^ may result in incomplete separation since LBs may be still associated. Pederson and colleagues compared proteins of cores prepared with NP-40/DTT to those isolated from infected cells treated with actinomycin D which inhibits uncoating. Only the NP40/DTT-treated cores were positive for the LB protein F17, which indicates incomplete LB removal by NP40/DTT treatment^25^. Although LBs may be partially removed by trypsin digestion^20^, no conclusion can be drawn which distinct viral proteins are ubiquitinated when analyzing core and membrane fraction by immunoblot. The LC-MS/MS-based ubiquitinome analysis performed in this study enabled us to map ubiquitination sites in viral proteins precisely, with diGly(K) residues providing direct evidence of ubiquitination.

The detection of diverse ubiquitin linkages in CPXV particles does not necessarily imply that these chains are covalently bound to a target protein, as free ubiquitin chains have been identified in cells^31,32^. Preformed ubiquitin chains can also be transferred to substrates by ubiquitin ligases^33^. However, Mercer and colleagues concluded from the molecular weight of K48-linked ubiquitin bands in immunoblot analysis that ubiquitin in VACV particles was substrate bound^4^.

Furthermore, the number of viral ubiquitination sites somewhat correlated with the ubiquitin amount in the virus particles (proteome analysis prior to diGly(K) enrichment; data not shown) and ranged from 247 in CPXV BR to 668 in CPXV HumGri which were assigned to 86 to 148 viral proteins (Table 1). This data indicates possible strain-specific differences in the CPXV ubiquitinome.

**Table 1:** Ubiquitin amount, viral ubiquitination sites and associated viral proteins.

CPXV strain	Ubiquitin [µmol/g protein]^a^	Number of viral diGly(K) sites	Number of viral diGly(K) proteins
BR	0.52	247	86
RatKre	0.57	403	117
RatHei	0.52	476	127
HumBer	0.61	487	131
HumGri	1.23	668	148

^a^Ubiquitin amount in non-diGly(K)-enriched virions calculated by total protein approach (TPA)^61^. By TPA method, protein concentrations can be estimated without spike-in standard, assuming that the spectral intensity is proportional to the protein abundance.

### The conserved CPXV mature virion ubiquitinome

We were interested in identifying conserved ubiquitination sites among the five analyzed CPXV strains, since these sites might be functionally more important than non-conserved sites. A list of all identified ubiquitination sites in each CPXV strain is provided in Supplementary Table S4. In total, 137 conserved ubiquitination sites were identified, considering also homologous lysine residues of proteins with sequence difference between strains. These sites were assigned to 54 CPXV proteins, all belonging to the conserved CPXV mature virion proteome except for A36 (Table 2 and Supplementary Table S5)^17^.

**Table 2:** CPXV mature virion proteins with conserved ubiquitination sites.

Gene CPXV^a^	Gene VACV^b^	Number of diGly(K) sites^c^	Protein description
A11L	129	10	Major core protein 4a precursor
A7L	125	7	Protein A6
A44R	167	6	Profilin
A4L	122	6	Major core protein 4b
E13L	118	6	Scaffold protein D13
H1L	078	6	Metalloendopeptidase G1
J1L	099	6	Tyr/ser protein phosphatase
L1L	070	6	Telomere-binding protein I1
N4R	091	6	Core protein VP8
A26L	148	5	A-type inclusion protein A25
H8L	085	5	Assembly protein G7
J3L	101	5	IMV heparin-binding surface protein
A24R	143	4	Intermediate transcription factor 3 large subunit
D12L	002	3	TNF alpha receptor-like protein
G13L	052	3	Envelope protein F13
G17R	056	3	LB phosphoprotein F17
H4L	081	3	Glutaredoxin-2
A14L	132	2	Virion membrane protein A13
C3L	199	2	Putative uncharacterized protein
C8L	013	2	Interleukin 18-binding protein
E5R	110	2	Primase D5/uncoating factor
F6R	062	2	Putative uncharacterized protein
F8R	064	2	Membrane protein, ass. with IV/IMV and core
H2L	079	2	Entry/fusion complex component protein
M1L	032	2	Interferon antagonist K1L
Q1L	028	2	Inhibitor of TNF-R and TLR signaling
S1R	093	2	Virion protein J1
A17L	136	1	Virion membrane protein A16
A19R	138	1	Transcript termination protein A18
A1L	119	1	Viral late gene transcription factor 2
A23R	142	1	DNA Holliday junction resolvase A22
A25R	144	1	DNA-directed RNA polymerase 133 kDa polypeptide
A28L	150	1	14 kDa cell fusion protein
A30L	152	1	DNA-directed RNA polymerase 35 kDa subunit
A35R	157	1	EEV glycoprotein
A36R	158	1	Inhibitor of MHC class II antigen presentation
A50L	173	1	Putative uncharacterized protein
A52R	175	1	Putative uncharacterized protein
A53R	176	1	DNA ligase
A6R	124	1	DNA-directed RNA polymerase 19 kDa subunit
A8L	126	1	Early transcription factor 82 kDa subunit
B2R	184	1	Putative uncharacterized protein
B4R	187	1	EEV type-I membrane glycoprotein
C17L	025	1	Complement control protein C3
F3L	059	1	Double-stranded RNA-binding protein
F4L	060	1	DNA-directed RNA polymerase 30 kDa polypeptide
H10R	087	1	Myristoylated protein G9
H3R	080	1	Late transcription elongation factor G2
H5R	082	1	Putative nuclease G5
L7L	076	1	Viral core cysteine proteinase
N1R	088	1	Myristoylated IMV virion protein
N3L	090	1	IMV protein
O1R	095	1	Cap-specific mRNA (nucleoside-2′-O-)-methyltransferase
O4R	098	1	DNA-directed RNA polymerase 147 kDa polypeptide

^a^according to CPXV GRI-90.

^b^according to VACV WR.

^c^conserved in five analyzed CPXV strains.

Assuming 152 viral proteins in CPXV mature virion particles^17^, we demonstrate that at least 35% of CPXV mature virion proteins are ubiquitinated at conserved lysine residues, underlining the overall importance of ubiquitination for OPV. Another eleven viral proteins contained ubiquitination sites in all five CPXV strains but no conserved lysine residues were identified (Supplementary Table S6). More than half of the 54 viral proteins with conserved ubiquitination sites contained one or two diGly(K) sites, while other proteins showed up to ten sites (Fig. 4b). As expected, proteins with conserved diGly(K) sites tended to be essential for virus replication (59.5%) and conserved in OPV (70.4%). In addition, most viral proteins with conserved ubiquitination sites belonged to the functional term host–virus interaction and transcription but also proteins belonging to the core were identified (Fig. 4c). The identification of ubiquitination sites in CPXV proteins of diverse functions indicated an underestimated regulatory function of ubiquitin during OPV infection. Because the elucidation of ubiquitination sites in CPXV core proteins was a major objective of the present study, it is discussed in the following. Nevertheless, remarkable results were obtained for viral proteins of other functional classes. Our data, for example, indicates a ubiquitin-dependent transcriptional regulation of CPXV. Therefore, viral proteins with conserved ubiquitination sites that belong to functional classes other than the core are depicted in the Supplementary Discussion.

### Ubiquitination of CPXV core proteins

Although proteins of the core are not defined in detail, they are known to include the viral proteins A11 (p4a), A4 (p4b), A5 (p39), N4 (p25) and A7 transferred by homology from VACV^25,34,35^. These proteins, except for A7, are also found in high copy numbers comparable to those of membrane proteins in CPXV mature virions, suggesting a structural role^17^. The function of A7 has not been elucidated yet. A7 and N4 are known to locate inside the core, while A11, A4 and A5 are part of the outer core structure, making them likely targets for ubiquitination^25,35^. The major core protein A11 contained the greatest number of ubiquitination sites among all proteins identified. A11 contained ten conserved diGly(K) sites and a mean of 20 diGly(K) sites, considering also non-conserved sites. Additionally, the major core protein A4 contained six conserved diGly(K) sites, ranking third-highest in the number of conserved ubiquitination sites. The A5 protein was not grouped to the proteins with conserved ubiquitin positions because no site was detected in the CPXV BR strain; all other CPXV strains showed at least one ubiquitination site in the A5 protein. Although N4 and A7 are not part of the outer core structure, they were identified to possess six and seven conserved ubiquitination sites, respectively, demonstrating that ubiquitination of core proteins is not restricted to the outer part of the core. This data clearly confirmed the massive ubiquitination of CPXV major core proteins, supporting the hypothesis of their proteasomal degradation upon infection.

Ubiquitination of CPXV mature virion proteins may be catalyzed by one or numerous cellular E3 ubiquitin ligases^36^ or by a viral-encoded ubiquitin ligase^14^. CPXV encode one E3 ubiquitin ligase, the C7 protein (p28), which catalyzes K63-linked polyubiquitination *in vitro*^15^. Also the accumulation of K48-linked polyubiquitin in VF has been shown to be promoted by p28^37^. Additionally, it is assumed that at least one cellular ubiquitin ligase recruits ubiquitin to VF^37^. P28 is expressed early in infection and locates with ubiquitin to VF^16,37^, making the K48-linked polyubiquitination of CPXV mature virion proteins catalyzed by p28 an attractive hypothesis. However, p28 has been shown to be essential only for replication of ectromelia virus^38^ in mouse macrophages, suggesting the compensative activity of another ubiquitin ligase in cells other than macrophages.

Summarized, our results confirm previously described ubiquitination sites, as depicted in more detail in the Supplementary Discussion. Furthermore, distinct ubiquitination sites were elucidated in viral proteins which have been associated with ubiquitin before, but whose modification sites were previously unknown. However, there are some discrepancies between known ubiquitination sites in VACV and those identified in CPXV that may reflect species-specific differences. For example, it has been reported that VACV C6 (CPXV C14) and B14 (CPXV B12) are not covalently ubiquitinated^39^. Indeed, for the CPXV BR strain we were unable to identify any ubiquitination site in C14 or B12, but all other analyzed CPXV strains contained ubiquitination sites on one or both proteins. Hence, ubiquitination may contribute to species- and strain-specific differences of OPV. As ubiquitination may also depend on the host, it possibly also contributes to OPV adaptation to new hosts.

### CPXV proteins do not contain a ubiquitination motif near target lysine residues

Having identified up to 668 ubiquitination sites in a single CPXV strain encouraged us to search for a primary structure motif. We compared the amino acid sequence around ubiquitinated lysine residues (+/− eight amino acids) to the non-ubiquitinated lysine background using motif-x^40^. However, this did not lead to any results. Also, the sequence logo analysis implemented in Perseus revealed no primary amino acid motif for ubiquitination. Therefore, it may be concluded that ubiquitination site specificity of CPXV mature virion proteins is independent of a distinct amino acid motif near target lysine residues. It is not completely understood what determines ubiquitination site specificity but recently it was suggested that proteins destined for the proteasome contain three factors which are referred to as tripartite degron architecture^41^. These consist of a primary peptide motif recognized by an E3-ubiquitin ligase (degron), target lysine site(s) for ubiquitination and a disordered degradation initiation site. The degron can be distant from the target ubiquitination site, which could explain why no ubiquitination motif was identified near the lysine residue.

Besides ubiquitination, phosphorylation of VACV mature virion proteins has been globally analyzed with LC-MS/MS^42,43^. Recently, 396 unique phosphorylation sites among 83 VACV proteins have been identified^43^. Strikingly, about 70% of CPXV proteins with conserved ubiquitination sites were identified to contain phosphorylation sites in homologous VACV proteins. This correlation may be important since cross-talk between phosphorylation and ubiquitination has been described, e.g. the regulation of proteasomal degradation by phosphorylation^44^. Interestingly, the data from Ngo and colleagues^43^ revealed the greatest number of phosphorylation sites in VACV core proteins p4A and p4B with 17 and 34 unique phosphorylation sites, respectively, indicating that core proteins are packaged in a phosphorylated state in mature virions. Since it is known that phosphorylation motifs, so-called phosphodegrons, can serve as recognition signals for E3 ligases^44^, it can be hypothesized that OPV proteins may contain phosphorylation motifs representing binding signals for ubiquitin ligases. Although Ngo and colleagues^43^ did not detect a phosphorylation motif in VACV proteins, they observed protein areas with frequent phosphorylation identifications, so-called phosphorylation clusters, which were also found in major core proteins p4A and p4B.

### UPS-dependent degradation of viral proteins early in infection

**Figure 5:**
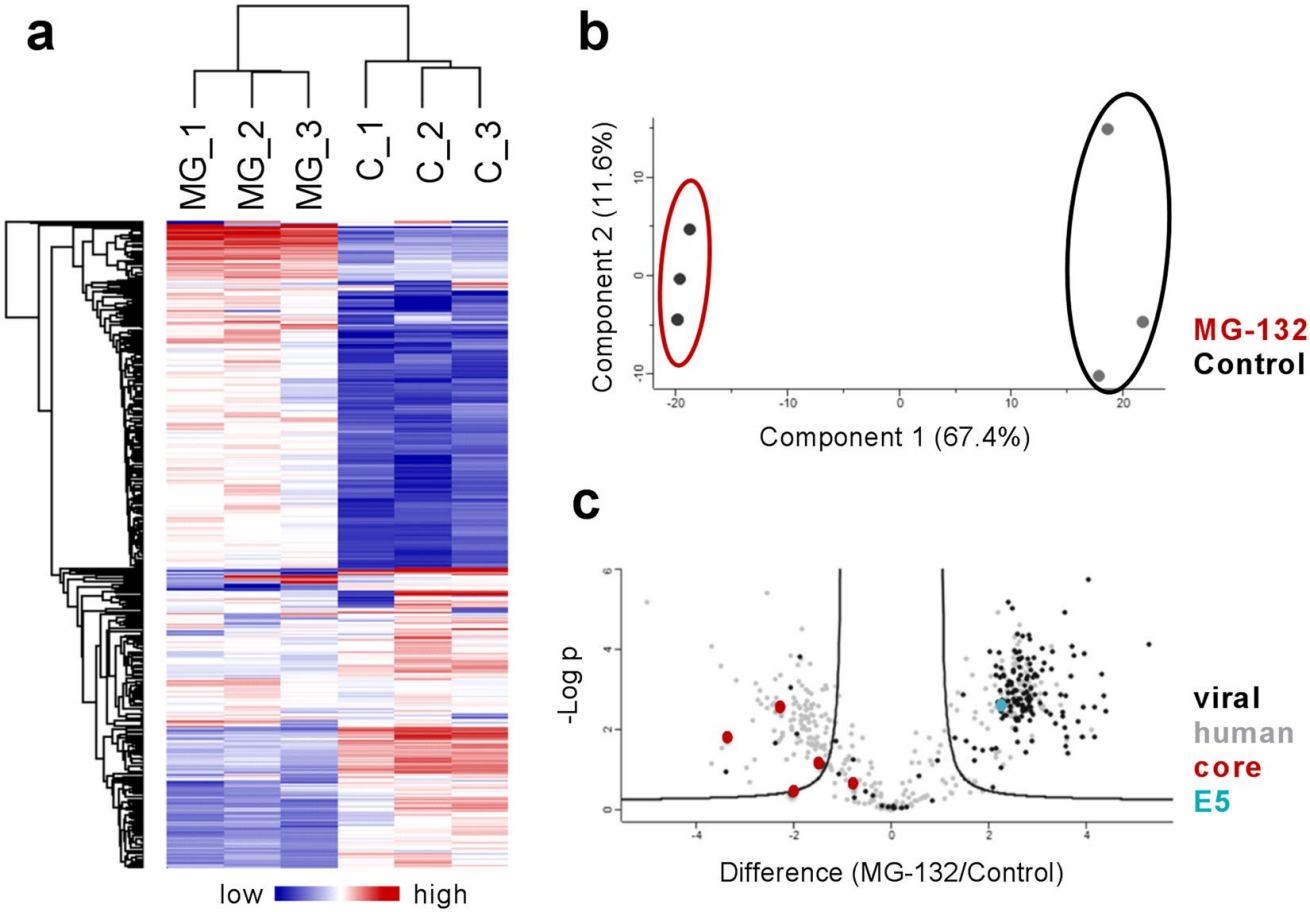
Proteasome-mediated degradation of ubiquitinated peptides at an early stage of CPXV infection. HeLa cells were infected in triplicate with CPXV BR at an MOI of 50 in the presence or absence of 10 µM MG-132. After 2 h post infection ubiquitinated peptides were analyzed by MS. Plots show normalized ubiquitinated peptide areas. (**a**) Cluster analysis reveals ubiquitinated peptides that are more abundant when the proteasome is inhibited (samples MG_1 to 3) compared to cells with non-inhibited proteasome (samples C_1 to 3), indicating their proteasomal degradation. (**b**) Inhibitor-treated samples (red) can clearly be separated from control samples (black) using principal component analysis. (**c**) Volcano plot showing significant differences (t-test, S0 = 2, permutation-based FDR 5%) in the amount of ubiquitinated peptides. Most viral peptides (black) are degraded in a proteasome-dependent manner but not major core proteins A11 and A4 (red). Instead, the uncoating factor E5 is proteasomally degraded.

As multiple ubiquitination sites in CPXV core proteins were elucidated, we next aimed to show their proteasome-dependent degradation in HeLa cells. For this purpose, we performed an LC-MS/MS-based quantitative analysis of ubiquitinated peptides in CPXV-infected HeLa cells in the presence or absence of MG-132 early in infection (2 h post infection). Control and inhibitor-treated samples were prepared in triplicate each, which show clear separation in hierarchical clustering and principal component analysis (Fig. 5a,b). Statistical analysis revealed significant abundance differences of ubiquitinated peptides in cells with non-inhibited versus inhibited proteasome, with most viral peptides being stabilized in the presence of proteasome inhibitor (Fig. 5c and Supplementary Table S7). Stabilization of proteins as a result of proteasome inhibition implies that these proteins are degraded by an active proteasome. Controversially, one peptide of the viral RhoA-signaling inhibitor G11 protein was identified to be degraded by the proteasome, while two peptides of G11 were identified as not being degraded. Since it cannot be inferred from the present data which ubiquitination linkage is present at the respective lysine linkage, the ubiquitin linkages in G11 may be not linked by K48 and, hence, may be not associated with proteasomal degradation. However, peptides of all other viral proteins showed clear tendencies for either degradation or not. Functional categorization of CPXV proteins degraded by the proteasome revealed mostly proteins of unknown function, followed by proteins belonging to host–virus interaction, transcription and DNA replication. Although proteins associated with host–virus interaction and transcription were also abundant among CPXV proteins with conserved ubiquitination sites, only few correlations between viral proteins degraded by the proteasome and conserved ubiquitination sites were observed (Supplementary Table S7). Out of 54 viral proteins degraded by the proteasome, 45 are part of the CPXV mature virion proteome^17^ but only 11 were identified with conserved ubiquitination site.

According to our hypothesis, we expected ubiquitinated peptides of CPXV major structural core proteins to accumulate upon proteasome inhibition. Surprisingly, we observed the opposite. Major core proteins A11 and A4 were unchanged or even less ubiquitinated when the proteasome was inhibited, disproving their direct proteasomal degradation. The outer core protein A5 was not identified in this experiment, which is consistent with the fact that no ubiquitination sites in A5 were identified in the ubiquitinome analysis in the CPXV BR strain used in this experiment. Besides structural core proteins, proteins that showed a downregulation upon MG-132 addition were primarily host proteins. Generally, the downregulation of ubiquitination upon proteasome inhibition may be catalyzed by deubiquitinating enzymes. It is not known how ubiquitination of proteins is altered during CPXV infection. However, since no deubiquitinating enzyme in CPXV has been described, host deubiquitinating enzymes may be involved.

Subsequently, we focused on the identification of CPXV proteins other than core proteins that could explain the inhibition of viral replication as a result of proteasome inhibition, and another class of viral proteins attracted our attention. Using siRNA screening, Kilcher and colleagues identified 15 viral candidate genes that impaired intermediate but not early VACV gene expression^23^. Out of these 15 candidate genes, four reduced the number of pre-replication sites, meaning precursor sites of viral DNA replication, by more than 40%. Strikingly, all of these four proteins were found to be stabilized by proteasome inhibition in our study. These proteins include the serine/threonine-protein kinase B1 (CPXV B1), the putative nuclease G5 (CPXV H5), the ssDNA-binding phosphoprotein I3 (CPXV L3) as well as the primase D5 (CPXV E5). Kilcher and colleagues identified the VACV D5 protein as the genome uncoating factor the depletion of which leads to accumulation of intact cores in the cytoplasm^23^. D5 has been shown to possess N-terminal primase as well as C-terminal NTPase activity *in vitro*^45–48^. While both activities are essential for viral DNA replication, only the latter is essential for uncoating^23^. D5 is expressed early during infection and recruited to VACV cores in cells with inhibited proteasome but not in cells with non-inhibited proteasome, indicating a temporary localization which is reversed by the proteasome^23^. CPXV E5 and VACV D5 share about 98.5% amino acid identity. In this study we provide evidence that the CPXV D5 homologue is ubiquitinated at two conserved lysine residues and degraded by the proteasome early in infection, supporting the hypothesis of a proteasomally regulated D5-core interaction. It has been proposed that D5 may assist uncoating by actively contributing to global core degradation or by acting at defined core channels^23^. Our data do not exclude the proteasome-dependent degradation of CPXV core proteins at a later stage of infection but indicate that the proteasomal degradation of other viral proteins, probably the uncoating factor D5 homologue, is a prerequisite. Other viral proteins that were identified to be proteasomally degraded early in infection are discussed in the supplements.

Besides 54 viral proteins, we found 151 ubiquitinated host proteins that significantly differed in abundance in response to proteasome inhibition of CPXV-infected cells (Supplementary Table S8). It should be noted that the identification of host proteins was not a major aim of this study. Hence, peptides were not fractionated prior to LC-MS/MS analysis as recommended for the detection of thousands of ubiquitination sites in cell lines^26^.

## Conclusions

The UPS plays an important role not only during OPV infection^2,3,23^ but also during infection of viruses belonging to diverse other families^5,6^, demonstrating the general regulatory function of ubiquitin in viral infection.

In this study, we present the first global ubiquitination site analysis of CPXV mature virion proteins, which is, moreover, the first published ubiquitinome analysis of any virus particles. The identified modification sites provide the basis for the elucidation of associated biological processes which we showed using the example of uncoating. In doing so, we disproved the hypothesized degradation of polyubiquitinated OPV core proteins immediately upon infection. Instead, we hypothesize from our data that the proteasomal reversion of the interaction between E5 and viral core proteins is a prerequisite for OPV genome uncoating.

Since the established protocol is applicable to analyze differences in the ubiquitination status of virus proteins during infection in a global approach, the present study provides the groundwork for future proteomic studies to elucidate the diverse roles of ubiquitin during viral infections.

## Methods

### Cells and viruses

HeLa cells (ATCC CCL-2) were grown in Eagle’s Minimal Essential Medium (EMEM) (Gibco) supplemented with 2 mM L-glutamine (PAA) and 10% (vol/vol) FCS (PAA). HEp-2 (ATCC#CCL-23) and Vero E6 cells (ECACC #84113001) were maintained in Dulbecco’s Modified Eagle Medium (DMEM) (Gibco) containing 2 mM L-Glutamine and 5% FCS. The following CPXV strains were used: Brighton Red (BR; NCBI GenBank #AF482758.2), RatHei09 (RatHei; NCBI GenBank #KC813504.1), RatKre08 (RatKre; NCBI GenBank #KC813505.1), HumGri07 (HumGri; NCBI GenBank #KC813511.1) and HumBer07 (HumBer; NCBI GenBank #KC813509.1). Recombinant CPXV expressing red fluorescent protein under an early and green fluorescent protein under a late viral promotor (CPXV BRFseR) was generously provided by Karsten Tischer (Freie Universität Berlin). CPXV proteins are named according to the GRI-90 strain (NCBI GenBank #X94355.2) and protein descriptions were obtained from UniProt.

### Purification of mature virions

HEp-2 cells were cultured in T175 cm^2^ cell culture flasks and infected with a multiplicity of infection (MOI) of 0.01–0.1. Four days post infection, cells were scraped in culture medium, pelleted and resuspended in 10 mM Tris pH 9.0. Cells were disrupted with glass beads and vigorous vortexing for 90 s. After sonication for 3 min, glass beads and cell debris were pelleted at 1,000 × *g* and 4 °C for 5 min. Purification of mature virions was done by rate-zonal sucrose gradient centrifugation^49^ in sterile SW 28 centrifuge tubes. For this purpose, the supernatant was centrifuged through a 36% sucrose cushion at 32,900 × *g* and 4 °C for 80 min and the virus pellet was resuspended in 1 ml of 10 mM Tris pH 9.0. The virus suspension was sonicated for 1 min, layered on a 24% to 40% continuous sucrose gradient and centrifuged at 26,000 × *g* and 4 °C for 50 min. The virus band was collected and stored at 4 °C, while the pellet was resuspended in 1 ml of 10 mM Tris pH 9.0, sonicated and centrifuged through a fresh 24% to 40% continuous sucrose gradient as described. The virus bands were pooled and concentrated at 32,900 × *g* and 4 °C for 60 min. The pellet was resuspended in 1 ml of 10 mM Tris pH 9.0 and stored in aliquots at −80 °C.

### Plaque assay

The number of PFU was determined by infecting 3 × 10^5^ Vero cells per well in a 24-well plate with 200 µl of virus suspension, using serial dilutions ranging from 10^−4^ to 10^−9^. Medium containing 1.6% (w/v) carboxymethyl cellulose was added 4 h post infection and cells were incubated for 4 days. Fixation of cells was done in 3.5% formaldehyde in PBS for 20 min, followed by 20 min of incubation with staining solution (0.1% (w/v) naphthol blue black, 1.36% (w/v) sodium acetate and 6% (v/v) acetic acid). PFU per ml were calculated using the mean of 4 wells of the same dilution.

### Treatment with proteasome inhibitors

MG-132 (Merck) and Bortezomib (New England Biolabs) were dissolved in dimethyl sulfoxide (DMSO), diluted with EMEM and sterile filtered through a 0.2 µm filter. If not stated otherwise, HeLa cells were treated with proteasome inhibitors simultaneously to infection. Control cells of all experiments were treated with the same amount of DMSO as inhibitor-treated cells.

### Analysis of proteasome activity

The chymotryptic proteasome activity was analyzed using the Proteasome 20 S Activity Assay (Sigma-Aldrich) according to manufacturer’s instructions. Briefly, 10,000 HeLa cells per well were seeded into a 96-well opaque black plate and incubated overnight. After three wash steps with PBS, cells were treated with 1 and 10 µM MG-132 or 0.01, 0.1 and 1 µM Bortezomib in medium without FCS, and the proteasome activity was measured 24 h post inhibitor addition. For this purpose, cells were incubated at 37 °C for 4 h with 100 µl/well of Proteasome Assay Loading Solution protected from light, and the fluorescence intensity was measured at 525 nm with excitation at 490 nm.

### Analysis of proteasome inhibitor-induced cytotoxicity

The release of lactate dehydrogenase (LDH) was measured using the LDH Cytotoxicity Assay Kit (Pierce) according to the Chemical Compound-Mediated Cytotoxicity Assay Protocol in the manufacturer’s instructions. In summary, 10,000 HeLa cells per well in a clear 96-well plate were treated with 10 µM MG-132 or 1 µM Bortezomib. After 24 h the LDH release was analyzed according to manual instructions.

### Analysis of cellular ATP content

The cellular ATP content was analyzed with the CellTiter-Glo® Luminescent Cell Viability Assay (Promega). Briefly, 10,000 HeLa cells per well in a 96-well white opaque plate were treated with 1 and 10 µM MG-132 or 0.01, 0.1 and 1 µM Bortezomib for 24 h and the ATP content was measured according to manufacturer’s instructions.

### Effect of proteasome inhibition on viral replication

HeLa cells in 24-well plates were infected with CPXV BR at an MOI of 1 in the presence or absence of 10 µM MG-132 or 1 µM Bortezomib. After incubation at 4 °C for 1 h the virus suspension was removed and cells were washed with PBS and incubated with or without proteasome inhibitors for 4 or 24 h. The supernatant was centrifuged at 1,000 × *g* for 5 min and assayed for genome equivalents (GE) and PFU.

### Confocal laser scanning microscopy

A total of 60,000 HeLa cells per well in a 24-well plate were seeded on sterile cover slips. Cells were infected with CPXV BRFseR at an MOI of 5 in the presence or absence of 10 µM MG-132 or 1 µM Bortezomib. After 8 h cells were washed with PBS and fixed for 1 h in 4% paraformaldehyde in PBS. After washing with PBS and water, cells were mounted with Mowiol/DAPI solution (Life Technologies). The coverslips were placed on clean microscopy slides and allowed to dry overnight protected from light. Fluorescence was observed with a confocal laser scanning microscope (LSM 780, Zeiss).

### Electron microscopy

HeLa cells were propagated in T25 cm^2^ cell culture flasks and pretreated with or without 10 µM MG-132 1 h prior to infection with CPXV BRFseR at an MOI of 100. After 4 h of incubation in the presence or absence of MG-132, cells were fixed in 2.5% glutaraldehyde in 0.05 M HEPES pH 7.2 for 2 h at room temperature. The samples were post fixed with OsO_4_ (1% in distilled water, 1 h), tannic acid (0.1% in Hepes 0.05 M, 30 min) and uranyl acetate (1% in distilled water, 2 h), followed by stepwise dehydration in a graded ethanol series and embedding in epon resin which was subsequently polymerized at 60 °C for 48 h^50^. Thin sections were produced with an ultramicrotome (UC7; Leica, Wetzlar, Germany) and counterstained with uranyl acetate and lead citrate. The sections were examined using a transmission electron microscope (Tecnai12; FEI) operated at 120 kV. For a quantification of intact virus cores and VF, 100 cells were randomly chosen and analyzed at the transmission electron microscope.

### Determination of viral GE

Viral DNA was isolated from 5 μL of purified virus suspension or from 200 µl of HeLa supernatant using the PureLink^®^ Viral RNA/DNA Mini Kit (Life Technologies) according to manufacturer’s instructions. DNA was eluted in 50 µl of RNase-free water and viral GE were quantified using an OPV real-time PCR assay^51^. By measuring plasmid standards in the range of 10^1^ to 10^6^ copies per reaction the amount of GE was calculated.

### Determination of protein concentration

The protein content was determined via tryptophan fluorescence measured with a microplate reader (Tecan Infinite® M1000 PRO)^17,52^. Briefly, 3 μL of lysate were mixed with 197 μL of 8 M urea in 50 mM Tris pH 8.5 (UA) and the fluorescence was measured at 350 nm with 295 nm excitation in a white opaque 96-well plate. The tryptophan content was determined by measuring a tryptophan standard curve ranging from 0.1 to 0.9 μg of tryptophan. Calculation of protein content was performed by assuming a tryptophan weight content of 1.3% for virus^17^ and 1.19% for cell lysates^52^.

### Virion lysis and filter-aided sample preparation (FASP)

Purified virus particles in 500 µl of 10 mM Tris pH 9.0 were pelleted at 25,000 × *g* and 4 °C for 30 min. Pellets were lysed in 120 μL of 4% SDS, 100 mM Tris pH 7.6, 10 mM Tris(2-carboxyethyl)phosphine and 40 mM 2-Chloroacetamide by heating at 95 °C for 5 min. The lysates were sonicated for 1 min, clarified at 16,000 × *g* for 5 min and prepared for MS analysis using a modified FASP method^53^. Briefly, 200 μg of protein were filled up with UA to 230 µl and loaded onto a Microcon Centrifugal Filter Unit with 30 kDa MWCO (Merck). Removal of SDS was achieved by washing 3 times with 200 μL of UA. Urea was replaced by washing 3 times with 50 mM ammonium bicarbonate (ABC) and digestion was performed overnight with Trypsin/Lys-C Mix (Promega) at 37 °C in 40 µl of ABC using an enzyme:protein ratio of 1:25. Tryptic peptides were recovered by centrifugation and washing twice with 40 μL of ABC. Peptides were desalted with 3 M Mili-SPE Extraction Disc Cartridges C18 SD (Sigma-Aldrich)^53^ and dried in a vacuum concentrator.

### Immunoaffinity purification (IAP) of ubiquitinated peptides

Enrichment of ubiquitinated peptides was performed with the PTMScan® Ubiquitin Remnant Motif (K-ε-GG) Kit (New England Biolabs) according to the manufacturer’s instructions. Briefly, desalted tryptic peptides were reconstituted in 1.4 ml of IAP buffer and incubated with washed motif antibody-bead slurry at 4 °C for 2 h. After 3 washes with IAP buffer and 3 washes with MS-grade water, diGly(K) peptides were eluted in 55 µl of 0.15% trifluoroacetic acid, followed by a second elution step with 50 µl of 0.15% trifluoroacetic acid. DiGly(K)-enriched peptides were desalted using 200 μl StageTips with two Empore^™^ SPE Disks C18 (3 M Purification)^54^ and concentrated in a vacuum concentrator.

### Effect of proteasome inhibition on ubiquitinated peptides in CPXV-infected HeLa cells

HeLa cells in T75 cm^2^ cell culture flasks were pretreated with or without 10 µM MG-132 at 37 °C for 1 h and infected with CPXV BR at an MOI of 50 in the presence or absence of proteasome inhibitor (triplicates). After 2 h post infection cells were scraped into medium and washed twice with PBS. Cell pellets were lysed and digested with FASP prior to enrichment of diGly(K) peptides analogous to virions.

### LC-MS/MS analysis

Analysis of diGly(K)-enriched peptides was performed with an Easy-nanoLC (Thermo Fisher Scientific) coupled to an LTQ Orbitrap Discovery mass spectrometer (Thermo Fisher Scientific). For single-run shotgun analysis, peptides were reconstituted in 0.1% formic acid and quantified by absorbance measurement at 280 nm using a Nanodrop (Thermo Fisher Scientific). Identical peptide amounts were loaded on a Reprosil-Pur 120 C18-AQ, 2.4 μm, 300 mm × 75 μm fused silica capillary column (Dr. Maisch) and separated with a linear 90 min gradient of acetonitrile in 0.1% formic acid and 3% DMSO from 0 to 40% at a flow rate of 200 nl/min. The column heater temperature was set to 60 °C and a spray voltage of 2.0 kV was applied. For background ion reduction an ABIRD device was used. The transfer capillary was heated to 275 °C without applying sheath or auxiliary gas flow. The orbitrap mass analyzer operated at a resolution of 30,000, scanning a mass range of 300–1,250 m/z. Data-dependent fragmentation of the 12 most intense ions with charge state ≥ 1 + was performed using CID fragmentation in the ion trap applying 35% normalized collision energy. The threshold for MS² spectra selection was set to 500 counts with maximally allowed ion accumulation times of 500 ms for full scan and 150 ms for fragment spectra. Viral peptides were analyzed in technical duplicates.

### MS data analysis

DiGly(K) peptides were identified with SEQUEST database search algorithm implemented in the Proteome Discoverer computational proteomics platform (v2.1). MS^2^ spectra of both technical duplicates were searched against the human UniProt complete proteome set with isoforms, the protein sequences of the respective CPXV strain and a contaminant database (cRAP). All CPXV databases were translated from the genome sequence (NCBI GenBank: BR #AF482758.2; Hei #KC813504.1; Kre #KC813505.1; Ber #KC813509.1 and Gri #KC813511.1). The enzyme specificity was set to trypsin (full) allowing for two missed cleavages. Mass tolerances were 10 ppm for parent ions and 0.6 Da for fragment ions. The maximum number of dynamic modifications per peptide was set to 4, including methionine oxidation, protein N-terminus acetylation and diGly modification of lysine residues. Cysteine carbamidomethylation was set as static modification. Peptides were identified with a false discovery rate (FDR) of 1% estimated by Percolator algorithm^55^, and peptide areas were calculated using the Precursor Ions Area Detector node.

Identification and label-free quantification of ubiquitinated peptides in HeLa cells was performed using the Andromeda^56^ and the MaxLFQ algorithm^57^, respectively, implemented in the MaxQuant computational proteomics platform (v1.5.1.2)^58^. MS^2^ spectra were searched against the human UniProt complete proteome set including isoforms, the CPXV BR database and a contaminant database applying specific trypsin/P digestion with a maximum of two missed cleavages. Variable and fixed modifications were set analogous to those of the virion peptide identification. Mass tolerances were 4.5 ppm for parent ions and 0.5 Da for fragment ions. A strict peptide FDR of 1% was applied while protein FDR was neglected. The match-between-runs option was used to transfer peptide identifications between samples within a match time window of 0.7 min and an alignment time window of 20 min. Further bioinformatics analysis of peptide identifications was done in Perseus (v1.5.0.31)^59^. Therefore, peptide raw intensities were filtered for reverse and contaminant peptide hits. Log_2_ transformed peptide intensities were normalized by median column subtraction and peptides with at least one diGly(K) modification were kept for further analysis. Categorically annotated control and inhibitor-treated replicates were filtered for at least three valid values in at least one group and normalized by median row subtraction. Missing values were imputed from normal distribution with default values mimicking low abundance measurements (width 0.3, down shift 1.8). Significant differences of ubiquitinated peptide quantities were analyzed using a two-sided t-test (S0 = 2) with 5% permutation-based FDR and 250 randomizations.

### Data availability

The MS proteomics data have been deposited to the ProteomeXchange Consortium via the PRIDE^60^ partner repository with the dataset identifier PXD006426.

## Electronic supplementary material

Supplementary Information
