# Supplementary material for: Global ubiquitination analysis reveals extensive modification and proteasomal degradation of cowpox virus proteins, but preservation of viral cores
Source: Sci Rep. 2018 Jan 29;8:1807. doi: 10.1038/s41598-018-20130-9 (PMC5788924; doi:10.1038/s41598-018-20130-9)
Supplement: Supplementary file 1 — Supplementary Information [file 41598_2018_20130_MOESM1_ESM.pdf]

Supplementary Information

Global ubiquitination analysis reveals extensive modification and proteasomal degradation of cowpox virus proteins, but preservation of viral cores

Marica Grossegesse[1], Joerg Doellinger[1,2,*], Annemarie Fritsch[1], Michael Laue[3], Janett Piesker[3], Lars Schaade[4], Andreas Nitsche[1]

Robert Koch Institute
[1]Centre for Biological Threats and Special Pathogens: Highly Pathogenic Viruses (ZBS 1), Berlin, 13353, Germany
[2]Centre for Biological Threats and Special Pathogens: Proteomics and Spectroscopy (ZBS 6), Berlin, 13353, Germany
[3]Centre for Biological Threats and Special Pathogens: Advanced Light and Electron Microscopy (ZBS 4), Berlin, 13353, Germany
[4]Centre for Biological Threats and Special Pathogens, Berlin, 13353, Germany

***Correspondence** should be sent to Joerg Doellinger, Robert Koch Institute, Centre for Biological Threats and Special Pathogens: Proteomics and Spectroscopy (ZBS 6), Seestr. 10, 13353 Berlin, Germany
doellingerj@rki.de; Phone +49 (0)30 18754 2373

Content:

A) Supplementary Discussion
1. CPXV proteins with conserved ubiquitination sites (except core proteins)
2. Viral proteins degraded by the proteasome

B) Supplementary Tables
Table S1: LC-MS/MS analysis of CPXV mature virion ubiquitinome: data overview.
Table S2: Origin of diGly(K) peptides.
Table S3: Quantification of lysine linkages using peptide areas.
Table S4: Identified ubiquitination sites in five CPXV strains.
Table S5: The conserved CPXV IMV ubiquitinome.
Table S6. Ubiquitinated CPXV mature virion proteins without conserved sites.
Table S7: CPXV proteins regulated by the proteasome.
Table S8: Host proteins degraded by the proteasome.

A) Supplementary discussion

1. CPXV proteins with conserved ubiquitination sites (except core proteins)

Host–virus interaction. Of the eleven ubiquitinated proteins assigned to the term host–virus interaction, six proteins are associated with viral immune evasion, including inhibitor of antigen presentation A36, complement control protein C17, IL-18-binding protein C8, inhibitor of TNF-R and TLR signaling Q1 (VACV N1) and two host range factors F3 (VACV E3) and M1 (VACV K1). Of those proteins, VACV E3 is known to be ubiquitinated but distinct lysine residues have not been identified yet[1]. Also, VACV N1 has been shown to be ubiquitinated at all six possible lysine residues[2]. In our study we were able to identify two conserved ubiquitination sites in the CPXV Q1 protein at positions 70 and 78 which are identical to those in VACV N1. In total, CPXV Q1 contains five lysine residues which are homologous to VACV N1, all of which were found to be ubiquitinated in CPXV HumBer and four of which found to be ubiquitinated in HumGri, RatKre and RatHei. The remaining five CPXV proteins assigned to host–virus interaction include A44, D12, G13, J1 (VACV VH1) and CPXV N1 protein. VH1 is a viral serine/threonine protein phosphatase which is located in LBs and requires a functional proteasome for activity, presumably to release the enzyme from the LBs[3]. We could show that J1, the CPXV homologue of VH1, contains six conserved ubiquitination positions, indicating an ubiquitin-dependent regulation of J1.

Transcription. The OPV DNA-directed RNA polymerase (Pol) is composed of eight subunits displaying similarity to the eukaryotic polymerase[4,5]. Especially, the two large subunits O4 and A25 display homology to the eukaryotic polymerase subunits[6]. Additionally, the F4 subunit shows homology to the eukaryotic transcription elongation factor SII[7]. Of the eight CPXV Pol subunits, five contain conserved ubiquitination sites including O4 (147 kDa), A25 (133 kDa), A30 (35 kDa), F4 (30 kDa) and A6 (19 kDa). The eukaryotic Pol II is known to become K63-polyubiquitinated following DNA damage, for example, and ubiquitination of Pol II in eukaryotes is also a mechanism of transcriptional regulation[8-10]. Considering the given homology between eukaryotic and OPV Pol, a similar regulation mechanism might also apply to OPV.

Besides subunits of the CPXV Pol itself, also viral transcription factors of all three classes contain ubiquitination sites. These include the A8 protein, a subunit of the early transcription factor, the A24 protein, an intermediate transcription factor subunit, and A1, a viral late transcription factor. Eukaryotic transcription factors seem to be activated and also tagged for degradation by ubiquitination[11,12], but no mechanism has been described for OPV yet. Furthermore, the late transcription elongation factor H3, the transcription termination protein A18 and the 2'-O-methyltransferase O1 possess conserved ubiquitination sites.

DNA replication. Ubiquitination is also involved in CPXV DNA replication[13]. The viral DNA ligase A53, Holliday junction resolvase A23 and the putative nuclease H5 contain conserved ubiquitination positions. Interestingly, the E5 protein, a homologue to the VACV D5 protein, which has been identified as the VACV genome uncoating factor[14], contains two conserved ubiquitination sites that might be important for uncoating activity as discussed later on.

Membrane. Two membrane proteins A14 and A17 as well as two EEV glycoproteins A35 and B4 contain conserved ubiquitination sites. Although purified IMV particles were analyzed and the co-purification of EEV particles seems unlikely because of their unstable outer membrane[15], it has been shown that EEV proteins can be detected in IMV virion preparations[16]. Furthermore, the scaffold protein E13 and the H2 protein belonging to the entry/fusion complex are ubiquitinated.

Virus entry into host cell. Three CPXV proteins associated with entry into the host cell were identified to be ubiquitinated. These proteins include the cell fusion protein A28, the IMV heparin-binding surface protein J3 and the myristoylated protein H10.

Morphogenesis. Proteins with conserved ubiquitination sites assigned to morphogenesis comprise the protein F8, metalloendopeptidase H1, assembly protein H8, telomere-binding protein L1 and the virion protein S1.

Unknown. Also ubiquitination sites on CPXV proteins A50, A52, A7, B2, C3 and F6 of unknown function were identified. A7 is known to locate in the core[17] containing, with 7 diGly(K) sites, the greatest number of ubiquitination sites in unknown CPXV proteins.

Protein processing, viral reproduction and others. This summarized functional term includes the H4 protein, G17 protein, L7 cysteine proteinase and A26 A-type inclusion protein. VACV homologs of the oxidoreductase H4 (Glutaredoxin) and the phosphoprotein G17 have been identified to be part of the LBs[3]. F17 is known to be degraded in a proteasome-dependent manner and suggested to be an LB scaffold protein [3]. The sequence of CPXV G17 is identical to that of the VACV homologue F17 except for amino acid position 83 and contains 7 lysine residues at positions 17, 25, 28, 41, 47, 50 and 74. Five distinct ubiquitination sites of VACV F17 on K25, K28, K41, K47 and K50 have been previously identified by MS[3]. Of those positions, we could identify K25 as a conserved ubiquitination site in CPXV and furthermore identified two additional conserved sites at K17 and K74. In contrast, we were not able to identify ubiquitination at K28 and K41 for CPXV.

2. Viral proteins degraded by the proteasome

Besides the proteasome-dependent degradation of proteins associated with reduction of prereplication sites[14], a notable number of viral BTB/kelch proteins were found to be regulated by the UPS. Cellular proteins of the BTB/kelch family act as adaptor proteins by an N-terminal BTB domain which interacts with cullin-3 ubiquitin ligase (CUL3) and a C-terminal

kelch domain recruiting the substrate to be targeted for proteasomal degradation[18,19]. CPXV code for six BTB/kelch proteins: A57, B9, B19, C18, D11 and G3. Our data demonstrates that all of the CPXV BTB/kelch proteins except for A57 are degraded in a proteasome-dependent manner early in infection. For A57, B19, C18 and G3 the interaction with the cellular CUL3-containing CRL complex has been shown[18], but so far no substrate of CPXV BTB/kelch proteins has been identified and the distinct role remains unclear. Mercer et al. reported that CUL3-mediated ubiquitination is required for VACV DNA replication[13], but as CPXV BTB/kelch proteins are not essential for CPXV replication[20], they are not likely to be the according adapter molecules in this context. However, deletion of CPXV BTB/kelch genes D11L, C18L, G3L and A57R has been shown to lead to altered host range and reduced virulence in mice, illustrating their importance in CPXV infection[21].

Furthermore, in the context of protein ubiquitination, CPXV encode 13 proteins belonging to the ANK/PRANC protein family which interact with the cellular cullin-1 ubiquitin ligase complex (SCF)[18]. These proteins are characterized by five to ten ankyrin repeats (ANK) at the N-terminus that mediate specific protein–protein interactions and a PRANC (C-terminal pox protein repeat of ankyrin C-terminus) domain similar to the cellular F-box domain[18,19]. By homology to the F-box domain, ANK/PRANC proteins are suspected to be substrate adaptors for SCF, although no substrate has been identified yet[18,19]. Additionally, the inhibition of SCF-mediated ubiquitination by binding of OPV ANK/PRANC proteins to SCF has been proposed[19]. Out of the 13 ANK/PRANC proteins, we identified five that are stabilized upon proteasome inhibition, indicating their proteasomal degradation in non-inhibitor-treated cells. These proteins include four proteins of unknown function (C1, C3, D14 and K1) as well as the 2'-O-methyltransferase O1. It has also been reported that siRNA-mediated knockdown of CUL-1 has no impact on VACV yield[13]. In agreement with this, CPXV ANK/PRANC proteins are not essential for replication[20] and rather may play a role in host range as shown for C9 (CP77)[22].

The presumed LB scaffold protein F17 (CPXV G17) is known to be degraded by the proteasome early in infection[3], but we were not able to show the degradation of G17. This was because we did not detect any diGly(K) peptide of G17 in our experiment with HeLa cells, although we previously detected three conserved ubiquitination sites in G17.

Moreover, we found that the CPXV ubiquitin p28-like ligase (C7) is degraded in a proteasome-dependent manner at an early stage of infection. P28 localizes with ubiquitin in VF[23,24] and is known to be regulated by autoubiquitination and proteasomal degradation[23], confirming our results.

References Supplementary Discussion

1. Gonzalez-Santamaria, J. *et al.* Regulation of Vaccinia Virus E3 Protein by Small Ubiquitin-Like Modifier Proteins. *J. Virol.* **85**, 12890-12900 (2011).
2. Maluquer de Motes, C., Schiffner, T., Sumner, R. P. & Smith, G. L. Vaccinia virus virulence factor N1 can be ubiquitylated on multiple lysine residues. *J Gen. Virol.* **95**, 2038-2049 (2014).
3. Schmidt, F. I. *et al.* Vaccinia virus entry is followed by core activation and proteasome-mediated release of the immunomodulatory effector VH1 from lateral bodies. *Cell Rep.* **4**, 464-476 (2013).
4. Knutson, B. A. & Broyles, S. S. Expansion of poxvirus RNA polymerase subunits sharing homology with corresponding subunits of RNA polymerase II. *Virus Genes* **36**, 307-311(2008).
5. Moss, B., Ahn, B. Y., Amegadzie, B., Gershon, P. D. & Keck, J. G. Cytoplasmic transcription system encoded by vaccinia virus. *J. Biol. Chem.* **266**, 1355-1358 (1991).
6. Ahn, B. Y., Gershon, P. D. & Moss, B. RNA polymerase-associated protein Rap94 confers promoter specificity for initiating transcription of vaccinia virus early stage genes. *J. Biol. Chem.* **269**, 7552-7557 (1994).
7. Ahn, B. Y., Gershon, P. D., Jones, E. V. & Moss, B. Identification of rpo30, a vaccinia virus RNA polymerase gene with structural similarity to a eucaryotic transcription elongation factor. *Mol. Cell. Biol.* **10**, 5433-5441 (1990).
8. Conaway, R. C., Brower, C. S. & Conaway, J. W. Emerging roles of ubiquitin in transcription regulation. *Science* **296**, 1254-1258 (2002).
9. Harreman, M. *et al.* Distinct ubiquitin ligases act sequentially for RNA polymerase II polyubiquitylation. *Proc. Natl. Acad. Sci. U. S. A.* **106**, 20705-20710 (2009).
10. Lee, K. B. & Sharp, P. A. Transcription-dependent polyubiquitination of RNA polymerase II requires lysine 63 of ubiquitin. *Biochemistry* **43**, 15223-15229 (2004).
11. Thomas, D. & Tyers, M. Transcriptional regulation: Kamikaze activators. *Curr. Biol.* **10**, R341-343 (2000).
12. Salghetti, S. E., Caudy, A. A., Chenoweth, J. G. & Tansey, W. P. Regulation of transcriptional activation domain function by ubiquitin. *Science* **293**, 1651-1653 (2001).
13. Mercer, J. *et al.* RNAi screening reveals proteasome- and Cullin3-dependent stages in vaccinia virus infection. *Cell Rep.* **2**, 1036-1047 (2012).
14. Kilcher, S. *et al.* siRNA screen of early poxvirus genes identifies the AAA+ ATPase D5 as the virus genome-uncoating factor. *Cell Host Microbe* **15**, 103-112 (2014).
15. Bidgood, S. R. & Mercer, J. Cloak and Dagger: Alternative Immune Evasion and Modulation Strategies of Poxviruses. *Viruses* **7**, 4800-4825 (2015).
16. Doellinger, J., Schaade, L. & Nitsche, A. Comparison of the Cowpox Virus and Vaccinia Virus Mature Virion Proteome: Analysis of the Species- and Strain-Specific Proteome. *PLoS One* **10**, e0141527 (2015).
17. Meng, X., Embry, A., Sochia, D. & Xiang, Y. Vaccinia virus A6L encodes a virion core protein required for formation of mature virion. *J. Virol.* **81**, 1433-1443 (2007).
18. Shchelkunov, S. N. Interaction of orthopoxviruses with the cellular ubiquitin-ligase system. *Virus Genes* **41**, 309-318 (2010).
19. Barry, M. *et al.* Poxvirus exploitation of the ubiquitin-proteasome system. *Viruses* **2**, 2356-2380 (2010).
20. Xu, Z., Zikos, D., Osterrieder, N. & Tischer, B. K. Generation of a complete single-gene knockout bacterial artificial chromosome library of cowpox virus and identification of its essential genes. *J. Virol.* **88**, 490-502, (2014).
21. Kochneva, G., Kolosova, I., Maksyutova, T., Ryabchikova, E. & Shchelkunov, S. Effects of deletions of kelch-like genes on cowpox virus biological properties. *Arch. Virol.* **150**, 1857-1870 (2005).
22. Spehner, D., Gillard, S., Drillien, R. & Kirn, A. A cowpox virus gene required for multiplication in Chinese hamster ovary cells. *J. Virol.* **62**, 1297-1304 (1988).
23. Mottet, K., Bareiss, B., Milne, C. D. & Barry, M. The poxvirus encoded ubiquitin ligase, p28, is regulated by proteasomal degradation and autoubiquitination. *Virology* **468-470**, 363-378 (2014).
24. Nerenberg, B. T. *et al.* The poxviral RING protein p28 is a ubiquitin ligase that targets ubiquitin to viral replication factories. *J. Virol.* **79**, 597-601 (2005).

B) Supplementary Tables

Table S1: LC-MS/MS analysis of CPXV mature virion ubiquitinome: data overview

Sample	MS/MS	PSMs	id [%]	Peptide groups	Protein groups	DiGly(K) peptides	Viral diGly(K) peptide ids[1]	Human diGly(K) peptide ids[a]
CPXV_BR	11925	3321	27.8	1940	882	889	249	352
CPXV_HumBer	11918	2804	23.5	1698	727	863	511	352
CPXV_HumGri	14443	4075	28.2	2270	916	1230	683	547
CPXV_RatHei	12088	3839	31.8	2254	951	726	491	235
CPXV_RatKre	14579	4336	29.7	2540	1149	656	410	246

[a] Contains redundant diGly(K) sites

Table S2: Origin of diGly(K) peptides

diGly peptide area [%][a]	Ber	Kre	BR	Gri	Hei	Mean	SD[b]
Viral	32.5	39.1	9.6	45.1	44.8	34.2	14.7
Human/contaminant	5.5	5.6	15.6	6.8	6.2	7.9	4.3
Ubiquitin	62.0	55.3	74.8	48.1	49.0	57.9	11.0

[a] % of total diGly(K) peptide area

[b] Standard deviation

Table S3: Quantification of lysine linkages using peptide areas

Lysine number	CPXV RatKre	CPXV BR	CPXV HumGri	CPXV RatHei	CPXV HumBer	Mean	Standard deviation
6	5.4	5.2	5.8	4.1	2.2	4.5	1.5
11	6.6	8.7	9.9	9.3	5.0	7.9	2.0
27	0.5	0.7	0.7	0.7	0.6	0.7	0.1
29	1.2	0.5	0.8	1.2	0.6	0.9	0.3
33	0.8	0.2	0.1	0.0	0.0	0.2	0.3
48	58.7	60.5	32.0	48.9	63.7	52.8	12.8
63	26.8	24.3	50.6	35.9	27.9	33.1	10.7

Table S4: Identified ubiquitination sites in five CPXV strains.

CPXV strain	Gene CPXV GRI-90	# PSMs	# Unique Peptides	diGly(K) count	diGly(K) Positions
CPXVHumBer	A11L	88	38	19	diGly [K149; K162; K163; K188; K193; K366; K369; K382; K384; K436; K462; K509; K558; K737; K757; K789; K795; K856; K876]
CPXVHumBer	A53R	22	13	15	diGly [K60; K64; K95; K184; K243; K256; K262; K267; K342; K377; K394; K452; K472; K475; K540]
CPXVHumBer	F6R	31	19	13	diGly [K90; K94; K112; K168; K287; K317; K372; K456; K460; K490; K552; K555; K556]
CPXVHumBer	A7L	51	17	12	diGly [K16; K86; K91; K136; K137; K194; K297; K299; K318; K321; K331; K353]
CPXVHumBer	A4L	74	26	11	diGly [K82; K83; K111; K164; K185; K189; K270; K311; K407; K471; K507]
CPXVHumBer	N4R	28	11	10	diGly [K127; K153; K170; K175; K202; K204; K207; K212; K240; K250]
CPXVHumBer	A26L	24	10	10	diGly [K15; K52; K77; K233; K238; K245; K246; K248; K254; K313]
CPXVHumBer	E13L	25	13	10	diGly [K172; K225; K231; K281; K354; K377; K419; K436; K469; K545]
CPXVHumBer	F2L	19	11	10	diGly [K64; K94; K103; K229; K238; K443; K480; K510; K523; K552]
CPXVHumBer	H1L	19	11	10	diGly [K7; K96; K109; K172; K369; K370; K478; K483; K584; K585]
CPXVHumBer	J4L	15	9	9	diGly [K16; K242; K300; K316; K516; K627; K643; K646; K667]
CPXVHumBer	A8L	18	12	9	diGly [K186; K205; K362; K381; K394; K468; K513; K641; K647]
CPXVHumBer	H8L	28	10	9	diGly [K36; K38; K99; K224; K246; K265; K268; K292; K350]
CPXVHumBer	L1L	22	11	9	diGly [K59; K64; K99; K100; K109; K174; K203; K232; K290]
CPXVHumBer	J1L	27	11	9	diGly [K8; K22; K40; K51; K53; K98; K159; K165; K170]
CPXVHumBer	A25R	13	10	8	diGly [K60; K116; K129; K139; K299; K452; K762; K800]
CPXVHumBer	A44R	36	9	7	diGly [K13; K16; K26; K29; K42; K59; K109]
CPXVHumBer	A24R	10	6	7	diGly [K139; K154; K283; K322; K324; K330; K357]
CPXVHumBer	M1L	15	8	7	diGly [K21; K242; K247; K253; K259; K262; K269]

CPXVHumBer	J3L	22	10	7	diGly [K39; K47; K109; K148; K164; K225; K254]
CPXVHumBer	A17L	16	7	6	diGly [K11; K113; K116; K133; K258; K278]
CPXVHumBer	C3L	18	9	6	diGly [K14; K60; K258; K287; K288; K666]
CPXVHumBer	B8R	10	6	6	diGly [K172; K174; K187; K197; K203; K217]
CPXVHumBer	A22R	13	5	6	diGly [K240; K243; K248; K306; K317; K349]
CPXVHumBer	B2R	10	6	6	diGly [K32; K142; K198; K240; K363; K370]
CPXVHumBer	E11L	12	7	6	diGly [K32; K221; K280; K341; K522; K597]
CPXVHumBer	H4L	17	7	6	diGly [K54; K81; K96; K99; K108; K113]
CPXVHumBer	S1R	12	6	6	diGly [K74; K82; K87; K114; K115; K146]
CPXVHumBer	G13L	29	9	6	diGly [K75; K168; K226; K231; K281; K368]
CPXVHumBer	E8L	9	6	5	diGly [K14; K32; K100; K211; K224]
CPXVHumBer	C1L	14	8	5	diGly [K185; K346; K363; K387; K398]
CPXVHumBer	Q1L	16	4	5	diGly [K25; K26; K70; K78; K117]
CPXVHumBer	L8R	8	7	5	diGly [K32; K152; K166; K364; K378]
CPXVHumBer	A27L	14	6	5	diGly [K36; K37; K61; K296; K297]
CPXVHumBer	F9L	5	5	5	diGly [K408; K642; K818; K844; K959]
CPXVHumBer	A47L	7	4	5	diGly [K45; K47; K151; K160; K296]
CPXVHumBer	M4L	8	6	5	diGly [K89; K300; K327; K380; K392]
CPXVHumBer	A35R	6	5	4	diGly [K111; K119; K125; K145]
CPXVHumBer	E5R	9	6	4	diGly [K124; K390; K647; K649]
CPXVHumBer	F8R	10	4	4	diGly [K13; K165; K180; K187]
CPXVHumBer	A28L	17	7	4	diGly [K37; K48; K98; K99]
CPXVHumBer	B11R	9	4	4	diGly [K49; K102; K159; K232]
CPXVHumBer	A13L	5	3	4	diGly [K61; K63; K88; K167]
CPXVHumBer	B1R	6	3	4	diGly [K62; K160; K162; K189]
CPXVHumBer	C8L	8	5	4	diGly [K66; K76; K93; K121]
CPXVHumBer	A19R	7	5	4	diGly [K81; K88; K290; K297]
CPXVHumBer	G16L	5	3	4	diGly [K92; K189; K230; K231]
CPXVHumBer	L7L	9	7	4	diGly [K97; K129; K155; K160]
CPXVHumBer	E1R	9	5	3	diGly [K107; K332; K797]
CPXVHumBer	C15L	7	3	3	diGly [K11; K13; K60]
CPXVHumBer	C9L	5	3	3	diGly [K112; K320; K454]
CPXVHumBer	F7R	8	3	3	diGly [K16; K69; K77]
CPXVHumBer	F1L	4	3	3	diGly [K166; K327; K451]

CPXVHumBer	G17R	15	5	3	diGly [K17; K25; K74]
CPXVHumBer	A12R	9	5	3	diGly [K171; K186; K190]
CPXVHumBer	E6R	3	3	3	diGly [K198; K213; K392]
CPXVHumBer	B3R	5	2	3	diGly [K205; K206; K266]
CPXVHumBer	H5R	6	4	3	diGly [K213; K282; K353]
CPXVHumBer	C18L	3	3	3	diGly [K29; K173; K486]
CPXVHumBer	F10R	4	3	3	diGly [K4; K84; K91]
CPXVHumBer	B4R	7	3	3	diGly [K41; K43; K259]
CPXVHumBer	A23R	6	3	3	diGly [K44; K149; K167]
CPXVHumBer	F3L	7	4	3	diGly [K45; K108; K176]
CPXVHumBer	A29L	5	2	3	diGly [K47; K70; K72]
CPXVHumBer	F4L	6	4	3	diGly [K60; K67; K80]
CPXVHumBer	G4L	5	3	3	diGly [K64; K158; K309]
CPXVHumBer	J2R	4	3	3	diGly [K66; K88; K117]
CPXVHumBer	H3R	3	2	2	diGly [K101; K107]
CPXVHumBer	J7R	3	2	2	diGly [K102; K135]
CPXVHumBer	B20R	3	2	2	diGly [K118; K274]
CPXVHumBer	B15L	2	2	2	diGly [K122; K282]
CPXVHumBer	O2R	2	1	2	diGly [K134; K135]
CPXVHumBer	M2L	2	2	2	diGly [K139; K270]
CPXVHumBer	A52R	5	2	2	diGly [K143; K148]
CPXVHumBer	A5L	7	4	2	diGly [K18; K27]
CPXVHumBer	N3L	7	6	2	diGly [K18; K272]
CPXVHumBer	G11L	3	2	2	diGly [K18; K315]
CPXVHumBer	E3R	3	2	2	diGly [K192; K231]
CPXVHumBer	H10R	2	2	2	diGly [K202; K236]
CPXVHumBer	B7R	2	2	2	diGly [K221; K228]
CPXVHumBer	O1R	10	7	2	diGly [K226; K234]
CPXVHumBer	E2L	4	2	2	diGly [K31; K146]
CPXVHumBer	O4R	4	2	2	diGly [K37; K109]
CPXVHumBer	A6R	4	2	2	diGly [K43; K70]
CPXVHumBer	A14L	12	4	2	diGly [K49; K56]
CPXVHumBer	A36R	6	2	2	diGly [K50; K64]
CPXVHumBer	B17R	2	2	2	diGly [K51; K202]
CPXVHumBer	I3R	3	3	2	diGly [K511; K542]
CPXVHumBer	A50L	3	2	2	diGly [K66; K83]
CPXVHumBer	B12R	3	2	2	diGly [K67; K104]
CPXVHumBer	H9R	7	4	2	diGly [K75; K180]
CPXVHumBer	E12L	4	2	2	diGly [K88; K218]
CPXVHumBer	H2L	3	2	2	diGly [K89; K111]
CPXVHumBer	D12L	5	3	2	diGly [K94; K106]
CPXVHumBer	B21R	3	1	1	diGly [K100]
CPXVHumBer	G1L	3	2	1	diGly [K103]
CPXVHumBer	A40L	1	1	1	diGly [K110]
CPXVHumBer	A34R	4	2	1	diGly [K123]

CPXVHumBer	Q2L	2	2	1	diGly [K126]
CPXVHumBer	E9R	2	1	1	diGly [K128]
CPXVHumBer	H7R	2	1	1	diGly [K132]
CPXVHumBer	A59R	18	7	1	diGly [K134]
CPXVHumBer	A3L	1	1	1	diGly [K15]
CPXVHumBer	A30L	4	1	1	diGly [K174]
CPXVHumBer	A2L	3	2	1	diGly [K175]
CPXVHumBer	A18L	3	1	1	diGly [K180]
CPXVHumBer	L3L	3	2	1	diGly [K182]
CPXVHumBer	J5R	3	2	1	diGly [K198]
CPXVHumBer	N1R	3	1	1	diGly [K21]
CPXVHumBer	F5R	2	1	1	diGly [K263]
CPXVHumBer	C16L	1	1	1	diGly [K271]
CPXVHumBer	A38R	1	1	1	diGly [K31]
CPXVHumBer	G15L	1	1	1	diGly [K31]
CPXVHumBer	G3L	3	2	1	diGly [K320]
CPXVHumBer	G5L	1	1	1	diGly [K321]
CPXVHumBer	E7R	1	1	1	diGly [K33]
CPXVHumBer	C6L	1	1	1	diGly [K331]
CPXVHumBer	B19R	1	1	1	diGly [K340]
CPXVHumBer	A1L	1	1	1	diGly [K36]
CPXVHumBer	G12L	2	2	1	diGly [K365]
CPXVHumBer	R1L	2	2	1	diGly [K366]
CPXVHumBer	A37R	1	1	1	diGly [K37]
CPXVHumBer	C14L	4	1	1	diGly [K49]
CPXVHumBer	A57R	1	1	1	diGly [K547]
CPXVHumBer	C17L	4	2	1	diGly [K62]
CPXVHumBer	A51R	2	1	1	diGly [K70]
CPXVHumBer	A58R	2	2	1	diGly [K72]
CPXVHumBer	B13R	1	1	1	diGly [K73]
CPXVHumBer	G10L	2	2	1	diGly [K79]
CPXVHumBer	M3L	1	1	1	diGly [K82]
CPXVHumBer	C7R	2	1	1	diGly [K84]
CPXVBR	A11L	93	33	19	diGly [K149; K162; K163; K189; K194; K367; K370; K383; K385; K437; K511; K560; K666; K739; K759; K791; K797; K803; K850]
CPXVBR	A4L	49	20	8	diGly [K82; K83; K111; K185; K189; K270; K311; K471]
CPXVBR	N4R	23	11	8	diGly [K153; K175; K202; K204; K207; K212; K240; K250]
CPXVBR	A26L	16	7	7	diGly [K15; K52; K77; K239; K246; K255; K314]
CPXVBR	A7L	20	8	7	diGly [K86; K136; K137; K194; K297; K299; K331]
CPXVBR	A44R	21	9	6	diGly [K13; K16; K26; K29; K42; K59]

CPXVBR	E13L	14	7	6	diGly [K172; K231; K354; K419; K436; K469]
CPXVBR	G13L	8	7	6	diGly [K68; K168; K226; K231; K355; K368]
CPXVBR	H1L	17	7	6	diGly [K7; K96; K109; K369; K370; K478]
CPXVBR	J1L	20	8	6	diGly [K22; K40; K51; K159; K165; K170]
CPXVBR	L1L	18	9	6	diGly [K59; K100; K109; K174; K203; K232]
CPXVBR	A24R	5	5	5	diGly [K139; K154; K283; K330; K357]
CPXVBR	A25R	10	7	5	diGly [K60; K116; K129; K139; K455]
CPXVBR	A53R	8	5	5	diGly [K21; K60; K377; K394; K469]
CPXVBR	C8L	7	5	5	diGly [K60; K66; K76; K93; K102]
CPXVBR	H8L	16	7	5	diGly [K36; K224; K246; K268; K292]
CPXVBR	J3L	15	8	5	diGly [K47; K109; K148; K225; K254]
CPXVBR	P1L	10	5	5	diGly [K47; K185; K198; K214; K321]
CPXVBR	A27L	11	4	4	diGly [K36; K37; K61; K93]
CPXVBR	E8L	12	7	4	diGly [K22; K100; K211; K224]
CPXVBR	F8R	11	6	4	diGly [K13; K165; K187; K267]
CPXVBR	J4L	8	5	4	diGly [K16; K242; K316; K577]
CPXVBR	A14L	14	5	3	diGly [K49; K56; K67]
CPXVBR	A19R	5	3	3	diGly [K81; K88; K464]
CPXVBR	A8L	8	5	3	diGly [K362; K513; K647]
CPXVBR	B7R	5	3	3	diGly [K135; K228; K241]
CPXVBR	D12L	10	3	3	diGly [K96; K108; K109]
CPXVBR	F6R	8	7	3	diGly [K90; K94; K445]
CPXVBR	G17R	14	5	3	diGly [K17; K25; K74]
CPXVBR	H10R	3	3	3	diGly [K230; K236; K308]
CPXVBR	H3R	3	3	3	diGly [K85; K107; K170]
CPXVBR	H4L	7	3	3	diGly [K54; K96; K99]
CPXVBR	H5R	4	4	3	diGly [K7; K282; K353]
CPXVBR	L7L	4	3	3	diGly [K53; K97; K129]
CPXVBR	M2L	5	3	3	diGly [K69; K139; K270]
CPXVBR	M4L	7	5	3	diGly [K86; K300; K327]
CPXVBR	S1R	4	3	3	diGly [K74; K82; K87]
CPXVBR	A17L	5	2	2	diGly [K11; K258]
CPXVBR	A29L	4	1	2	diGly [K70; K72]
CPXVBR	A35R	3	2	2	diGly [K111; K125]
CPXVBR	A52R	3	2	2	diGly [K143; K148]
CPXVBR	B4R	2	2	2	diGly [K41; K241]
CPXVBR	C3L	2	1	2	diGly [K273; K274]

CPXVBR	D1L	4	2	2	diGly [K129; K216]
CPXVBR	D6L	3	3	2	diGly [K4; K10]
CPXVBR	E5R	3	2	2	diGly [K124; K647]
CPXVBR	G4L	3	2	2	diGly [K138; K295]
CPXVBR	G7L	3	1	2	diGly [K21; K22]
CPXVBR	H2L	2	2	2	diGly [K89; K111]
CPXVBR	J7R	5	2	2	diGly [K102; K135]
CPXVBR	M1L	3	2	2	diGly [K21; K262]
CPXVBR	O2R	2	1	2	diGly [K134; K135]
CPXVBR	P2L	2	2	2	diGly [K121; K123]
CPXVBR	Q1L	5	2	2	diGly [K70; K78]
CPXVBR	A1L	2	1	1	diGly [K36]
CPXVBR	A23R	1	1	1	diGly [K44]
CPXVBR	A28L	2	2	1	diGly [K37]
CPXVBR	A30L	4	1	1	diGly [K174]
CPXVBR	A34R	1	1	1	diGly [K87]
CPXVBR	A36R	4	1	1	diGly [K64]
CPXVBR	A47L	1	1	1	diGly [K296]
CPXVBR	A50L	1	1	1	diGly [K83]
CPXVBR	A51R	1	1	1	diGly [K181]
CPXVBR	A58R	1	1	1	diGly [K66]
CPXVBR	A6R	1	1	1	diGly [K70]
CPXVBR	B14R	2	1	1	diGly [K203]
CPXVBR	B17R	1	1	1	diGly [K45]
CPXVBR	B2R	1	1	1	diGly [K370]
CPXVBR	B3R	1	1	1	diGly [K266]
CPXVBR	B6R	3	2	1	diGly [K178]
CPXVBR	B8R	1	1	1	diGly [K222]
CPXVBR	C12L	2	1	1	diGly [K128]
CPXVBR	C17L	3	2	1	diGly [K62]
CPXVBR	C1L	1	1	1	diGly [K396]
CPXVBR	C2L	2	1	1	diGly [K132]
CPXVBR	C9L	1	1	1	diGly [K465]
CPXVBR	E2L	2	1	1	diGly [K138]
CPXVBR	F3L	1	1	1	diGly [K45]
CPXVBR	F4L	4	2	1	diGly [K62]
CPXVBR	G11L	1	1	1	diGly [K315]
CPXVBR	H9R	5	3	1	diGly [K73]
CPXVBR	K1R	3	1	1	diGly [K474]
CPXVBR	N1R	2	1	1	diGly [K21]
CPXVBR	N3L	8	4	1	diGly [K272]
CPXVBR	O1R	5	4	1	diGly [K234]
CPXVBR	O4R	2	2	1	diGly [K37]
CPXVHumGri	A11L	113	39	23	diGly [K149; K162; K163; K188; K193; K366; K369; K382; K384;

					K436; K460; K462; K509; K558; K572; K737; K755; K789; K795; K801; K848; K856; K877]
CPXVHumGri	C9L	40	20	20	diGly [K20; K32; K64; K112; K132; K252; K289; K320; K383; K388; K454; K462; K537; K577; K578; K615; K633; K639; K658; K660]
CPXVHumGri	A7L	62	20	15	diGly [K16; K86; K91; K99; K136; K137; K189; K194; K297; K299; K318; K321; K331; K349; K353]
CPXVHumGri	A8L	32	17	14	diGly [K15; K54; K186; K205; K362; K381; K393; K394; K427; K467; K468; K513; K641; K647]
CPXVHumGri	E13L	39	16	14	diGly [K17; K172; K225; K231; K281; K354; K363; K364; K377; K419; K429; K436; K469; K545]
CPXVHumGri	F6R	45	20	14	diGly [K90; K94; K107; K112; K168; K317; K372; K404; K456; K460; K478; K490; K552; K556]
CPXVHumGri	A26L	33	17	13	diGly [K9; K15; K52; K77; K81; K233; K238; K245; K246; K248; K254; K313; K465]
CPXVHumGri	A25R	28	16	12	diGly [K3; K60; K109; K116; K129; K139; K165; K299; K390; K452; K455; K882]
CPXVHumGri	A4L	81	27	12	diGly [K82; K83; K111; K153; K164; K185; K189; K270; K311; K407; K471; K507]
CPXVHumGri	G13L	50	16	12	diGly [K68; K70; K75; K110; K111; K168; K226; K231; K281; K355; K356; K368]
CPXVHumGri	L1L	33	15	12	diGly [K19; K59; K64; K93; K99; K100; K109; K117; K129; K174; K203; K232]
CPXVHumGri	M1L	31	12	12	diGly [K10; K21; K75; K78; K205; K242; K247; K253; K259; K262; K269; K271]
CPXVHumGri	H8L	33	12	11	diGly [K36; K38; K224; K240; K246; K268; K278; K292; K344; K350; K364]
CPXVHumGri	O4R	19	11	11	diGly [K37; K109; K324; K603; K958; K972; K974; K1118; K1119; K1210; K1228]
CPXVHumGri	F2L	23	10	10	diGly [K64; K103; K229; K289; K475; K480; K510; K523; K552; K707]
CPXVHumGri	J4L	26	11	10	diGly [K16; K162; K163; K242; K245; K300; K316; K516; K643; K646]
CPXVHumGri	A53R	18	9	9	diGly [K60; K95; K262; K342; K377; K384; K394; K452; K540]

CPXVHumGri	B11R	18	8	9	diGly [K13; K14; K33; K49; K102; K139; K159; K162; K232]
CPXVHumGri	F9L	16	9	9	diGly [K408; K435; K642; K650; K734; K818; K844; K901; K959]
CPXVHumGri	H1L	21	10	9	diGly [K7; K96; K109; K369; K370; K420; K478; K483; K488]
CPXVHumGri	J3L	30	12	9	diGly [K39; K47; K54; K109; K148; K162; K225; K254; K267]
CPXVHumGri	N4R	28	12	9	diGly [K127; K153; K170; K175; K202; K207; K212; K240; K250]
CPXVHumGri	A17L	20	9	8	diGly [K11; K22; K113; K116; K133; K194; K258; K278]
CPXVHumGri	A22R	15	7	8	diGly [K18; K240; K243; K248; K306; K310; K317; K349]
CPXVHumGri	B12R	17	8	8	diGly [K48; K54; K63; K67; K77; K83; K87; K331]
CPXVHumGri	C3L	28	12	8	diGly [K14; K60; K223; K260; K289; K290; K369; K668]
CPXVHumGri	G10L	13	7	8	diGly [K79; K81; K239; K330; K355; K356; K408; K412]
CPXVHumGri	J1L	27	11	8	diGly [K8; K22; K40; K51; K98; K159; K165; K170]
CPXVHumGri	A13L	10	7	7	diGly [K22; K28; K61; K63; K88; K155; K167]
CPXVHumGri	A44R	38	9	7	diGly [K13; K16; K26; K29; K42; K59; K109]
CPXVHumGri	A47L	17	5	7	diGly [K45; K47; K149; K151; K160; K296; K340]
CPXVHumGri	C1L	25	10	7	diGly [K183; K344; K359; K361; K372; K385; K396]
CPXVHumGri	E5R	19	10	7	diGly [K36; K124; K291; K390; K435; K647; K649]
CPXVHumGri	L7L	17	9	7	diGly [K90; K97; K126; K129; K139; K146; K155]
CPXVHumGri	A24R	13	6	6	diGly [K153; K154; K283; K322; K330; K357]
CPXVHumGri	B1R	12	5	6	diGly [K160; K162; K189; K204; K238; K247]
CPXVHumGri	B8R	18	8	6	diGly [K172; K174; K187; K197; K203; K217]
CPXVHumGri	E11L	14	7	6	diGly [K32; K122; K266; K280; K334; K522]
CPXVHumGri	E1R	12	8	6	diGly [K107; K332; K433; K635; K730; K797]
CPXVHumGri	F4L	8	6	6	diGly [K55; K61; K68; K81; K92; K131]
CPXVHumGri	H4L	22	7	6	diGly [K54; K81; K96; K99; K108; K113]
CPXVHumGri	S1R	12	7	6	diGly [K74; K82; K83; K87; K114; K146]
CPXVHumGri	A19R	7	5	5	diGly [K81; K88; K170; K297; K464]

CPXVHumGri	A1L	6	4	5	diGly [K36; K91; K113; K115; K126]
CPXVHumGri	A28L	11	6	5	diGly [K37; K48; K57; K98; K99]
CPXVHumGri	B22R	6	5	5	diGly [K58; K1308; K1569; K1680; K1744]
CPXVHumGri	F8R	12	6	5	diGly [K13; K180; K187; K201; K267]
CPXVHumGri	G4L	10	5	5	diGly [K7; K50; K138; K269; K295]
CPXVHumGri	H5R	9	5	5	diGly [K7; K65; K213; K282; K353]
CPXVHumGri	M2L	6	5	5	diGly [K69; K139; K185; K194; K270]
CPXVHumGri	N1R	9	4	5	diGly [K21; K113; K125; K127; K217]
CPXVHumGri	A23R	10	4	4	diGly [K44; K55; K149; K167]
CPXVHumGri	A2L	5	4	4	diGly [K116; K167; K175; K179]
CPXVHumGri	A33L	6	4	4	diGly [K46; K145; K273; K296]
CPXVHumGri	A35R	15	6	4	diGly [K111; K119; K125; K130]
CPXVHumGri	B4R	6	2	4	diGly [K41; K43; K259; K262]
CPXVHumGri	B7R	7	3	4	diGly [K228; K230; K238; K241]
CPXVHumGri	C15L	11	5	4	diGly [K11; K13; K60; K76]
CPXVHumGri	C17L	6	3	4	diGly [K60; K62; K233; K235]
CPXVHumGri	C8L	14	6	4	diGly [K66; K76; K93; K102]
CPXVHumGri	E2L	6	4	4	diGly [K38; K58; K138; K146]
CPXVHumGri	E3R	5	3	4	diGly [K184; K192; K231; K232]
CPXVHumGri	G17R	29	8	4	diGly [K17; K25; K50; K74]
CPXVHumGri	H10R	8	4	4	diGly [K202; K217; K236; K308]
CPXVHumGri	H9R	13	6	4	diGly [K73; K75; K180; K215]
CPXVHumGri	L8R	9	6	4	diGly [K32; K152; K166; K242]
CPXVHumGri	N3L	12	7	4	diGly [K14; K18; K114; K272]
CPXVHumGri	Q1L	21	4	4	diGly [K25; K26; K70; K78]
CPXVHumGri	A12R	10	5	3	diGly [K170; K185; K189]
CPXVHumGri	A14L	21	6	3	diGly [K49; K56; K67]
CPXVHumGri	A27L	14	5	3	diGly [K36; K37; K296]
CPXVHumGri	A29L	4	2	3	diGly [K47; K70; K72]
CPXVHumGri	A3L	8	4	3	diGly [K6; K15; K65]
CPXVHumGri	A51R	7	2	3	diGly [K37; K40; K70]
CPXVHumGri	A57R	4	3	3	diGly [K137; K430; K541]
CPXVHumGri	B2R	6	3	3	diGly [K240; K363; K370]
CPXVHumGri	B9R	4	3	3	diGly [K301; K390; K478]
CPXVHumGri	C19L	4	2	3	diGly [K116; K145; K146]
CPXVHumGri	D12L	7	3	3	diGly [K94; K106; K107]
CPXVHumGri	E12L	6	3	3	diGly [K83; K88; K218]
CPXVHumGri	E6R	5	3	3	diGly [K198; K213; K392]
CPXVHumGri	F7R	8	3	3	diGly [K16; K70; K78]
CPXVHumGri	J2R	6	3	3	diGly [K66; K81; K94]
CPXVHumGri	J7R	5	3	3	diGly [K5; K102; K135]
CPXVHumGri	L6L	3	2	3	diGly [K6; K274; K275]

CPXVHumGri	M4L	7	4	3	diGly [K327; K380; K392]
CPXVHumGri	O1R	10	6	3	diGly [K226; K234; K235]
CPXVHumGri	O2R	3	2	3	diGly [K7; K134; K135]
CPXVHumGri	R1L	6	5	3	diGly [K317; K366; K524]
CPXVHumGri	A18L	2	1	2	diGly [K180; K187]
CPXVHumGri	A30L	5	2	2	diGly [K116; K174]
CPXVHumGri	A36R	5	2	2	diGly [K49; K63]
CPXVHumGri	A37R	2	2	2	diGly [K37; K42]
CPXVHumGri	A50L	3	2	2	diGly [K75; K83]
CPXVHumGri	A52R	8	2	2	diGly [K143; K148]
CPXVHumGri	A55R	2	1	2	diGly [K102; K104]
CPXVHumGri	A59R	19	8	2	diGly [K18; K134]
CPXVHumGri	A6R	5	3	2	diGly [K43; K70]
CPXVHumGri	B13R	3	2	2	diGly [K73; K96]
CPXVHumGri	B15L	2	2	2	diGly [K126; K282]
CPXVHumGri	B17R	3	2	2	diGly [K92; K202]
CPXVHumGri	B20R	4	3	2	diGly [K118; K274]
CPXVHumGri	B3R	4	2	2	diGly [K261; K266]
CPXVHumGri	E8L	9	4	2	diGly [K22; K211]
CPXVHumGri	E9R	4	2	2	diGly [K93; K128]
CPXVHumGri	F10R	6	2	2	diGly [K84; K91]
CPXVHumGri	F11L	5	3	2	diGly [K56; K122]
CPXVHumGri	F1L	2	2	2	diGly [K166; K451]
CPXVHumGri	F3L	7	3	2	diGly [K45; K108]
CPXVHumGri	G11L	5	2	2	diGly [K18; K315]
CPXVHumGri	H2L	5	2	2	diGly [K89; K111]
CPXVHumGri	H3R	2	2	2	diGly [K107; K209]
CPXVHumGri	H7R	3	2	2	diGly [K127; K132]
CPXVHumGri	M5L	2	2	2	diGly [K175; K254]
CPXVHumGri	M6R	5	2	2	diGly [K57; K71]
CPXVHumGri	A32R	4	2	1	diGly [K13]
CPXVHumGri	A34R	5	2	1	diGly [K86]
CPXVHumGri	A38R	1	1	1	diGly [K31]
CPXVHumGri	A41R	1	1	1	diGly [K165]
CPXVHumGri	A42R	1	1	1	diGly [K48]
CPXVHumGri	A54R	3	2	1	diGly [K40]
CPXVHumGri	A58R	4	2	1	diGly [K66]
CPXVHumGri	A5L	8	4	1	diGly [K27]
CPXVHumGri	B14R	1	1	1	diGly [K203]
CPXVHumGri	B16R	2	2	1	diGly [K503]
CPXVHumGri	B18R	1	1	1	diGly [K550]
CPXVHumGri	B19R	2	1	1	diGly [K340]
CPXVHumGri	B21R	2	1	1	diGly [K100]
CPXVHumGri	B6R	6	3	1	diGly [K179]
CPXVHumGri	C11L	1	1	1	diGly [K603]

CPXVHumGri	C14L	3	1	1	diGly [K49]
CPXVHumGri	C16L	1	1	1	diGly [K271]
CPXVHumGri	C18L	2	1	1	diGly [K486]
CPXVHumGri	C6L	1	1	1	diGly [K331]
CPXVHumGri	E4R	1	1	1	diGly [K86]
CPXVHumGri	F5R	4	1	1	diGly [K263]
CPXVHumGri	G12L	3	2	1	diGly [K365]
CPXVHumGri	G15L	2	1	1	diGly [K31]
CPXVHumGri	G16L	2	1	1	diGly [K92]
CPXVHumGri	G1L	3	2	1	diGly [K100]
CPXVHumGri	G3L	4	3	1	diGly [K320]
CPXVHumGri	G5L	1	1	1	diGly [K314]
CPXVHumGri	K1R	6	2	1	diGly [K475]
CPXVHumGri	L3L	2	1	1	diGly [K182]
CPXVHumGri	M3L	2	1	1	diGly [K82]
CPXVHumGri	N2R	2	1	1	diGly [K13]
CPXVHumGri	Q2L	4	3	1	diGly [K126]
CPXVHumGri	R2L	1	1	1	diGly [K49]
CPXVRatHei	A11L	165	54	21	diGly [K149; K162; K163; K188; K193; K366; K369; K382; K384; K436; K447; K460; K462; K509; K558; K664; K737; K789; K795; K848; K877]
CPXVRatHei	A7L	46	19	14	diGly [K16; K86; K91; K97; K99; K136; K137; K189; K194; K297; K299; K321; K331; K353]
CPXVRatHei	A25R	37	21	12	diGly [K16; K60; K109; K116; K123; K139; K165; K179; K452; K455; K652; K800]
CPXVRatHei	L1L	33	16	12	diGly [K19; K59; K64; K93; K99; K100; K109; K117; K174; K203; K232; K272]
CPXVRatHei	F2L	23	13	12	diGly [K64; K79; K81; K103; K229; K238; K435; K443; K480; K510; K523; K552]
CPXVRatHei	F6R	46	21	12	diGly [K90; K94; K112; K287; K317; K372; K456; K460; K490; K552; K555; K556]
CPXVRatHei	E13L	26	12	11	diGly [K172; K225; K231; K281; K354; K364; K377; K419; K436; K469; K545]
CPXVRatHei	N4R	47	14	10	diGly [K127; K153; K170; K175; K202; K204; K207; K212; K240; K250]
CPXVRatHei	A8L	28	13	9	diGly [K54; K186; K205; K362; K381; K513; K602; K641; K647]
CPXVRatHei	A4L	85	29	8	diGly [K111; K153; K185; K189; K270; K311; K471; K564]
CPXVRatHei	J4L	12	10	8	diGly [K16; K242; K245; K300;

					K316; K516; K577; K643]
CPXVRatHei	J1L	37	11	8	diGly [K22; K40; K51; K53; K98; K159; K165; K170]
CPXVRatHei	R1L	16	9	8	diGly [K24; K86; K231; K317; K363; K366; K444; K524]
CPXVRatHei	H8L	27	11	8	diGly [K36; K99; K210; K224; K246; K268; K292; K350]
CPXVRatHei	H1L	24	13	8	diGly [K7; K96; K109; K369; K370; K478; K483; K585]
CPXVRatHei	A44R	42	9	7	diGly [K13; K16; K26; K29; K42; K59; K109]
CPXVRatHei	A26L	18	8	7	diGly [K15; K52; K77; K238; K245; K254; K313]
CPXVRatHei	E11L	19	13	7	diGly [K32; K122; K266; K275; K280; K334; K522]
CPXVRatHei	G10L	13	9	7	diGly [K55; K79; K139; K329; K330; K355; K412]
CPXVRatHei	A53R	12	8	7	diGly [K60; K95; K256; K262; K342; K377; K394]
CPXVRatHei	M4L	21	9	7	diGly [K86; K89; K298; K300; K327; K380; K392]
CPXVRatHei	C9L	9	6	6	diGly [K112; K293; K387; K458; K466; K619]
CPXVRatHei	M1L	10	6	6	diGly [K21; K247; K253; K259; K262; K269]
CPXVRatHei	A27L	21	7	6	diGly [K36; K37; K61; K93; K106; K108]
CPXVRatHei	O4R	18	12	6	diGly [K37; K663; K972; K974; K1271; K1278]
CPXVRatHei	J3L	31	15	6	diGly [K38; K46; K108; K147; K224; K253]
CPXVRatHei	H4L	16	6	6	diGly [K54; K81; K96; K99; K108; K113]
CPXVRatHei	S1R	16	5	6	diGly [K82; K83; K87; K114; K115; K146]
CPXVRatHei	A17L	13	7	5	diGly [K11; K116; K133; K258; K278]
CPXVRatHei	E5R	15	9	5	diGly [K124; K390; K435; K647; K649]
CPXVRatHei	F8R	12	7	5	diGly [K13; K165; K180; K187; K267]
CPXVRatHei	A24R	10	6	5	diGly [K139; K154; K283; K330; K357]
CPXVRatHei	E6R	7	5	5	diGly [K198; K213; K392; K573; K575]
CPXVRatHei	B2R	11	6	5	diGly [K32; K240; K363; K370; K425]
CPXVRatHei	C3L	13	9	5	diGly [K60; K293; K294; K495; K672]
CPXVRatHei	F9L	5	5	5	diGly [K642; K734; K818; K844; K959]

CPXVRatHei	G13L	13	8	5	diGly [K68; K168; K226; K231; K281]
CPXVRatHei	E1R	13	10	4	diGly [K107; K332; K591; K635]
CPXVRatHei	G17R	30	7	4	diGly [K17; K25; K50; K74]
CPXVRatHei	Q1L	16	4	4	diGly [K25; K26; K70; K78]
CPXVRatHei	E2L	8	5	4	diGly [K31; K38; K58; K138]
CPXVRatHei	A23R	6	4	4	diGly [K44; K55; K149; K167]
CPXVRatHei	G4L	7	4	4	diGly [K7; K50; K269; K295]
CPXVRatHei	B1R	10	4	4	diGly [K71; K160; K162; K189]
CPXVRatHei	H9R	12	6	4	diGly [K73; K75; K180; K215]
CPXVRatHei	A12R	8	4	3	diGly [K120; K175; K190]
CPXVRatHei	J5R	7	4	3	diGly [K16; K140; K194]
CPXVRatHei	F7R	10	3	3	diGly [K16; K63; K78]
CPXVRatHei	N3L	17	9	3	diGly [K18; K114; K272]
CPXVRatHei	A5L	17	7	3	diGly [K18; K27; K33]
CPXVRatHei	H10R	5	3	3	diGly [K202; K236; K308]
CPXVRatHei	E8L	15	9	3	diGly [K22; K41; K108]
CPXVRatHei	A28L	14	8	3	diGly [K37; K48; K99]
CPXVRatHei	A51R	3	3	3	diGly [K37; K70; K181]
CPXVRatHei	F3L	7	3	3	diGly [K45; K108; K109]
CPXVRatHei	A33L	6	3	3	diGly [K46; K273; K283]
CPXVRatHei	A29L	8	2	3	diGly [K47; K70; K72]
CPXVRatHei	A14L	14	6	3	diGly [K49; K56; K67]
CPXVRatHei	F4L	5	4	3	diGly [K61; K81; K92]
CPXVRatHei	A50L	3	3	3	diGly [K75; K83; K227]
CPXVRatHei	A19R	7	5	3	diGly [K81; K88; K297]
CPXVRatHei	C7R	7	3	3	diGly [K84; K160; K236]
CPXVRatHei	H3R	4	3	3	diGly [K85; K107; K177]
CPXVRatHei	D12L	5	2	3	diGly [K94; K106; K107]
CPXVRatHei	L7L	13	6	3	diGly [K97; K129; K160]
CPXVRatHei	A55R	2	1	2	diGly [K102; K104]
CPXVRatHei	A35R	6	3	2	diGly [K119; K125]
CPXVRatHei	B15L	2	2	2	diGly [K122; K282]
CPXVRatHei	C15L	4	2	2	diGly [K13; K58]
CPXVRatHei	Q2L	5	3	2	diGly [K134; K135]
CPXVRatHei	A52R	6	2	2	diGly [K143; K148]
CPXVRatHei	A3L	3	2	2	diGly [K15; K45]
CPXVRatHei	F11L	4	3	2	diGly [K16; K122]
CPXVRatHei	A2L	2	2	2	diGly [K170; K175]
CPXVRatHei	A30L	5	3	2	diGly [K176; K299]
CPXVRatHei	A59R	18	8	2	diGly [K18; K134]
CPXVRatHei	D1L	4	3	2	diGly [K181; K220]
CPXVRatHei	E3R	4	3	2	diGly [K184; K192]
CPXVRatHei	D3L	7	5	2	diGly [K208; K542]
CPXVRatHei	N1R	4	2	2	diGly [K21; K217]

CPXVRatHei	O1R	22	12	2	diGly [K226; K234]
CPXVRatHei	B3R	3	2	2	diGly [K261; K266]
CPXVRatHei	F1L	4	3	2	diGly [K304; K451]
CPXVRatHei	L8R	13	8	2	diGly [K32; K152]
CPXVRatHei	C19L	2	2	2	diGly [K33; K114]
CPXVRatHei	A1L	3	2	2	diGly [K36; K91]
CPXVRatHei	B4R	3	1	2	diGly [K41; K43]
CPXVRatHei	L4L	8	3	2	diGly [K414; K655]
CPXVRatHei	G3L	5	4	2	diGly [K42; K320]
CPXVRatHei	A6R	6	3	2	diGly [K43; K70]
CPXVRatHei	A47L	5	1	2	diGly [K45; K47]
CPXVRatHei	M6R	2	2	2	diGly [K45; K59]
CPXVRatHei	B16R	4	3	2	diGly [K503; K506]
CPXVRatHei	C8L	2	2	2	diGly [K66; K76]
CPXVRatHei	O2R	4	2	2	diGly [K7; K160]
CPXVRatHei	H2L	4	2	2	diGly [K89; K111]
CPXVRatHei	H5R	4	3	2	diGly [K99; K282]
CPXVRatHei	J7R	1	1	1	diGly [K102]
CPXVRatHei	A10L	4	2	1	diGly [K112]
CPXVRatHei	D6L	1	1	1	diGly [K113]
CPXVRatHei	H7R	2	1	1	diGly [K132]
CPXVRatHei	K2R	1	1	1	diGly [K152]
CPXVRatHei	A13L	3	2	1	diGly [K167]
CPXVRatHei	G11L	1	1	1	diGly [K18]
CPXVRatHei	L3L	1	1	1	diGly [K182]
CPXVRatHei	M5L	2	2	1	diGly [K188]
CPXVRatHei	E12L	2	2	1	diGly [K218]
CPXVRatHei	F5R	1	1	1	diGly [K263]
CPXVRatHei	B14R	1	1	1	diGly [K280]
CPXVRatHei	A22R	1	1	1	diGly [K306]
CPXVRatHei	A38R	1	1	1	diGly [K31]
CPXVRatHei	G15L	1	1	1	diGly [K31]
CPXVRatHei	C6L	2	1	1	diGly [K331]
CPXVRatHei	B19R	2	1	1	diGly [K340]
CPXVRatHei	B18R	1	1	1	diGly [K472]
CPXVRatHei	K1R	2	1	1	diGly [K476]
CPXVRatHei	C14L	3	1	1	diGly [K49]
CPXVRatHei	A57R	1	1	1	diGly [K547]
CPXVRatHei	D4L	5	2	1	diGly [K564]
CPXVRatHei	O3L	1	1	1	diGly [K58]
CPXVRatHei	A36R	4	1	1	diGly [K64]
CPXVRatHei	C17L	3	2	1	diGly [K64]
CPXVRatHei	D14L	1	1	1	diGly [K645]
CPXVRatHei	J2R	1	1	1	diGly [K66]
CPXVRatHei	M3L	1	1	1	diGly [K82]

CPXVRatHei	A34R	2	2	1	diGly [K86]
CPXVRatHei	G16L	1	1	1	diGly [K92]
CPXVRatKre	A11L	125	50	17	diGly [K149; K188; K193; K366; K436; K447; K462; K509; K558; K572; K664; K737; K757; K789; K795; K848; K856]
CPXVRatKre	A7L	40	18	16	diGly [K16; K86; K91; K97; K99; K105; K136; K137; K189; K194; K201; K297; K299; K317; K321; K331]
CPXVRatKre	C9L	26	12	13	diGly [K64; K112; K287; K318; K381; K421; K422; K452; K460; K576; K613; K631; K637]
CPXVRatKre	L1L	41	17	12	diGly [K19; K59; K99; K100; K109; K117; K174; K178; K193; K203; K232; K254]
CPXVRatKre	A4L	100	32	11	diGly [K82; K83; K111; K153; K164; K185; K189; K270; K311; K407; K471]
CPXVRatKre	J3L	40	15	10	diGly [K38; K46; K77; K108; K116; K147; K161; K224; K253; K266]
CPXVRatKre	N4R	32	12	9	diGly [K127; K170; K175; K202; K204; K207; K212; K240; K250]
CPXVRatKre	E13L	21	11	8	diGly [K172; K231; K354; K364; K377; K419; K436; K469]
CPXVRatKre	F6R	34	19	8	diGly [K90; K94; K112; K317; K372; K456; K490; K556]
CPXVRatKre	J1L	34	12	8	diGly [K8; K22; K40; K51; K98; K159; K165; K170]
CPXVRatKre	A44R	41	9	7	diGly [K13; K16; K26; K29; K42; K59; K109]
CPXVRatKre	B1R	8	5	7	diGly [K70; K71; K160; K162; K189; K204; K238]
CPXVRatKre	E8L	28	15	7	diGly [K14; K22; K41; K108; K109; K211; K224]
CPXVRatKre	G13L	25	10	7	diGly [K68; K75; K168; K226; K231; K281; K368]
CPXVRatKre	H8L	27	11	7	diGly [K36; K38; K99; K224; K246; K268; K292]
CPXVRatKre	A24R	14	7	6	diGly [K139; K153; K154; K283; K330; K357]
CPXVRatKre	A47L	19	6	6	diGly [K45; K47; K149; K160; K296; K340]
CPXVRatKre	B19R	9	5	6	diGly [K7; K42; K50; K76; K80; K340]
CPXVRatKre	B22R	9	7	6	diGly [K58; K1297; K1558; K1669; K1733; K1894]
CPXVRatKre	H1L	19	10	6	diGly [K7; K96; K109; K369; K370; K478]
CPXVRatKre	H4L	14	7	6	diGly [K54; K81; K96; K99; K108; K113]

CPXVRatKre	A26L	9	5	5	diGly [K15; K77; K238; K245; K254]
CPXVRatKre	B2R	14	8	5	diGly [K142; K240; K363; K370; K425]
CPXVRatKre	C15L	9	5	5	diGly [K10; K12; K26; K59; K75]
CPXVRatKre	C19L	11	5	5	diGly [K33; K114; K143; K144; K158]
CPXVRatKre	C3L	18	9	5	diGly [K52; K60; K285; K286; K487]
CPXVRatKre	E5R	11	6	5	diGly [K124; K390; K647; K649; K745]
CPXVRatKre	G17R	28	9	5	diGly [K17; K25; K47; K50; K74]
CPXVRatKre	I5R	14	7	5	diGly [K138; K185; K186; K217; K225]
CPXVRatKre	M1L	6	4	5	diGly [K21; K253; K262; K269; K271]
CPXVRatKre	A53R	4	4	4	diGly [K182; K241; K260; K392]
CPXVRatKre	E12L	8	4	4	diGly [K83; K88; K95; K218]
CPXVRatKre	F2L	8	7	4	diGly [K64; K229; K443; K523]
CPXVRatKre	H9R	7	5	4	diGly [K73; K75; K180; K215]
CPXVRatKre	O2R	5	3	4	diGly [K7; K134; K135; K160]
CPXVRatKre	Q1L	21	4	4	diGly [K25; K26; K70; K78]
CPXVRatKre	S1R	8	3	4	diGly [K82; K87; K114; K115]
CPXVRatKre	A14L	14	6	3	diGly [K49; K56; K67]
CPXVRatKre	A17L	7	3	3	diGly [K116; K133; K258]
CPXVRatKre	A23R	5	3	3	diGly [K44; K149; K167]
CPXVRatKre	A32R	11	3	3	diGly [K13; K25; K27]
CPXVRatKre	A35R	6	3	3	diGly [K111; K119; K125]
CPXVRatKre	A3L	7	4	3	diGly [K15; K48; K65]
CPXVRatKre	A5L	16	8	3	diGly [K27; K33; K275]
CPXVRatKre	A8L	17	9	3	diGly [K186; K205; K513]
CPXVRatKre	C8L	6	4	3	diGly [K66; K76; K93]
CPXVRatKre	D12L	7	2	3	diGly [K94; K106; K107]
CPXVRatKre	E2L	6	5	3	diGly [K38; K138; K146]
CPXVRatKre	F3L	7	3	3	diGly [K45; K108; K109]
CPXVRatKre	F4L	9	5	3	diGly [K61; K68; K92]
CPXVRatKre	F9L	3	3	3	diGly [K21; K642; K818]
CPXVRatKre	G10L	7	5	3	diGly [K59; K239; K330]
CPXVRatKre	G4L	5	3	3	diGly [K7; K50; K295]
CPXVRatKre	H10R	5	3	3	diGly [K202; K230; K236]
CPXVRatKre	H5R	7	4	3	diGly [K213; K282; K353]
CPXVRatKre	P2L	5	4	3	diGly [K41; K123; K130]
CPXVRatKre	A13L	6	3	2	diGly [K28; K166]
CPXVRatKre	A19R	5	3	2	diGly [K88; K297]
CPXVRatKre	A22R	2	2	2	diGly [K18; K349]
CPXVRatKre	A25R	16	9	2	diGly [K139; K455]
CPXVRatKre	A28L	11	5	2	diGly [K37; K48]
CPXVRatKre	A30L	7	4	2	diGly [K71; K174]

CPXVRatKre	A33L	3	2	2	diGly [K46; K273]
CPXVRatKre	A36R	6	2	2	diGly [K50; K64]
CPXVRatKre	A42R	3	2	2	diGly [K48; K85]
CPXVRatKre	A50L	2	2	2	diGly [K66; K83]
CPXVRatKre	A55R	3	1	2	diGly [K102; K104]
CPXVRatKre	A6R	7	3	2	diGly [K43; K70]
CPXVRatKre	B21R	8	3	2	diGly [K98; K164]
CPXVRatKre	C1L	3	2	2	diGly [K363; K398]
CPXVRatKre	C6L	2	2	2	diGly [K7; K331]
CPXVRatKre	D11L	4	2	2	diGly [K181; K196]
CPXVRatKre	E1R	9	6	2	diGly [K107; K332]
CPXVRatKre	F7R	4	2	2	diGly [K16; K78]
CPXVRatKre	F8R	5	3	2	diGly [K13; K187]
CPXVRatKre	G16L	4	2	2	diGly [K92; K207]
CPXVRatKre	G3L	9	5	2	diGly [K320; K480]
CPXVRatKre	H2L	2	2	2	diGly [K89; K111]
CPXVRatKre	H3R	4	2	2	diGly [K107; K170]
CPXVRatKre	J4L	6	4	2	diGly [K577; K643]
CPXVRatKre	K1R	8	3	2	diGly [K216; K476]
CPXVRatKre	L4L	5	2	2	diGly [K414; K691]
CPXVRatKre	N1R	3	2	2	diGly [K21; K113]
CPXVRatKre	O1R	17	12	2	diGly [K226; K234]
CPXVRatKre	O4R	8	5	2	diGly [K37; K603]
CPXVRatKre	A12R	4	3	1	diGly [K171]
CPXVRatKre	A1L	2	1	1	diGly [K36]
CPXVRatKre	A2L	2	2	1	diGly [K175]
CPXVRatKre	A34R	3	2	1	diGly [K86]
CPXVRatKre	A38R	1	1	1	diGly [K31]
CPXVRatKre	A52R	4	1	1	diGly [K143]
CPXVRatKre	A57R	1	1	1	diGly [K430]
CPXVRatKre	B4R	4	2	1	diGly [K41]
CPXVRatKre	C14L	2	1	1	diGly [K49]
CPXVRatKre	C16L	2	1	1	diGly [K270]
CPXVRatKre	C17L	3	2	1	diGly [K58]
CPXVRatKre	C18L	2	2	1	diGly [K486]
CPXVRatKre	D6L	3	1	1	diGly [K10]
CPXVRatKre	E11L	13	6	1	diGly [K597]
CPXVRatKre	E3R	1	1	1	diGly [K192]
CPXVRatKre	E6R	2	2	1	diGly [K573]
CPXVRatKre	E9R	1	1	1	diGly [K128]
CPXVRatKre	F1L	3	2	1	diGly [K451]
CPXVRatKre	F5R	1	1	1	diGly [K263]
CPXVRatKre	G11L	3	1	1	diGly [K315]
CPXVRatKre	G12L	4	2	1	diGly [K121]
CPXVRatKre	G15L	2	1	1	diGly [K31]

CPXVRatKre	G1L	2	2	1	diGly [K81]
CPXVRatKre	I3R	1	1	1	diGly [K543]
CPXVRatKre	J2R	2	1	1	diGly [K66]
CPXVRatKre	L7L	6	3	1	diGly [K97]
CPXVRatKre	L8R	8	4	1	diGly [K32]
CPXVRatKre	M2L	5	3	1	diGly [K270]
CPXVRatKre	M4L	5	4	1	diGly [K380]
CPXVRatKre	N3L	14	7	1	diGly [K277]
CPXVRatKre	O3L	1	1	1	diGly [K92]
CPXVRatKre	Q2L	5	3	1	diGly [K126]

Table S5: The conserved CPXV IMV ubiquitinome.

Gene name (CPXV GRI-90)	Gene name (CPXV BR)	Gene name (VACV WR)	DiGly(K) positions in protein (CPXV BR)
A11L	CPXV142	VACWR129	149; 189; 194; 367; 437; 511; 560; 739; 791; 797
A14L	CPXV145	VACWR132	49; 56
A17L	CPXV149	VACWR136	258
A19R	CPXV151	VACWR138	88
A1L	CPXV132	VACWR119	36
A23R	CPXV155	VACWR142	44
A24R	CPXV156	VACWR143	154; 283; 330; 357
A25R	rpo132 gene	VACWR144	139
A26L	ati gene	VACWR148	15; 77; 239; 246; 255
A28L	CPXV162	VACWR150	37
A30L	CPXV164	VACWR152	174
A35R	CPXV169	VACWR157	125
A36R	CPXV171	VACWR158	64
A44R	CPXV179	VACWR167	13; 16; 26; 29; 42; 59
A4L	CPXV135	VACWR122	111; 185; 189; 270; 311; 471
A50L	CPXV185	VACWR173	83
A52R	CPXV187	VACWR175	143
A53R	CPXV188	VACWR176	394
A6R	CPXV137	VACWR124	70
A7L	CPXV138	VACWR125	86; 136; 137; 194; 297; 299; 331
A8L	CPX139	VACWR126	513
B2R	CPXV197	VACWR184	370
B4R	CPXV199	VACWR187	41
C17L	CPXV034	VACWR025	62
C3L	CPXV019	VACWR199	273; 274
C8L	CPXV024	VACWR013	66; 76
D12L	CPXV014	VACWR002	96; 108; 109
E13L	CPXV131	VACWR118	172; 231; 354; 419; 436; 469
E5R	CPXV122	VACWR110	124; 647
F3L	CPXV069	VACWR059	45
F4L	CPXV070	VACWR060	62
F6R	CPXV072	VACWR062	90; 94
F8R	CPXV074	VACWR064	13; 187
G13L	CPXV061	VACWR052	168; 226; 231
G17R	CPXV066	VACWR056	17; 25; 74
H10R	CPXV098	VACWR087	236
H1L	CPXV088	VACWR078	7; 96; 109; 369; 370; 478
H2L	CPX089	VACWR079	89; 111
H3R	CPXV090	VACWR080	107
H4L	CPXV091	VACWR081	54; 96; 99
H5R	CPXV092	VACWR082	282

H8L	CPXV095	VACWR085	36; 224; 246; 268; 292
J1L	CPXV110	VACWR099	22; 40; 51; 159; 165; 170
J3L	CPXV112	VACWR101	47; 109; 148; 225; 254
L1L	CPXV080	VACWR070	59; 100; 109; 174; 203; 232
L7L	CPXV086	VACWR076	97
M1L	CPXV041	VACWR032	21; 262
N1R	CPXV099	VACWR088	21
N3L	CPXV101	VACWR090	272
N4R	CPXV102	VACWR091	175; 202; 207; 212; 240; 250
O1R	CPXV106	VACWR095	234
O4R	CPXV109	VACWR098	37
Q1L	CPXV037	VACWR028	70; 78
S1R	CPXV104	VACWR093	82; 78

Table S6. Ubiquitinated CPXV mature virion proteins without conserved sites.

Gene CPXV[a]	Gene VACV[b]	Protein description
A34R	156	EEV glycoprotein
A47L	170	3β-Hydroxysteroid dehydrogenase/Δ 5-4 isomerase
C9L	014 (truncated)	Ankyrin repeat domain-containing protein
E2L	107	Core protein D2
E8L	113	Cell surface-binding protein
G11L	050	RhoA signaling inhibitor
G4L	043	Ribonucleoside-diphosphate reductase small chain
H9R	086	Late transcription factor 1
J4L	102	RNA polymerase-associated protein
M4L	035	Phospholipase-D-like protein K4
O2R	096	DNA-directed RNA polymerase 22 kDa subunit

[a] Gene name according to CPXV GRI-90 [b] Gene name according to VACV WR

Table S7: CPXV proteins regulated by the proteasome.

Gene (CPXV GRI-90)	Degradation by proteasome	Description
A22R	+	DNA polymerase processivity factor component A20
A24R	+	Intermediate transcription factor 3 large subunit
A25R	+	DNA-directed RNA polymerase 133 kDa polypeptide
A36R	+	Protein A35 Inhibitor of MHC class II antigen presentation
A38R	+	Putative uncharacterized protein
A49R	+	Toll/IL1-receptor [TIR]-like protein
A51R	+	Thymidylate kinase
A52R	+	Putative uncharacterized protein
A53R	+	DNA ligase
A54R	+	Putative uncharacterized protein
B11R	+	Probable serine/threonine-protein kinase B12
B12R	+	Serine proteinase inhibitor 2/CrmA
B19R	+	Kelch repeat and BTB domain-containing protein 2
B1R	+	Serine/threonine-protein kinase 1
B20R	+	Serine proteinase inhibitor 1
B22R	+	Putative uncharacterized protein
B2R	+	Putative uncharacterized protein
B9R	+	Kelch repeat protein B10
C16L	+	Putative uncharacterized protein
C18L	+	Kelch repeat protein C2
C1L	+	Putative uncharacterized protein
C3L	+	Putative uncharacterized protein
C6L	+	Protein C10
C7R	+	E3 ubiquitin-protein ligase p28-like
D10L	+	Putative uncharacterized protein
D11L	+	Kelch repeat/ BTB domain protein
D14L	+	Putative uncharacterized protein
D6L	+	Protein N2
E12L	+	mRNA-capping enzyme regulatory subunit
E1R	+	mRNA-capping enzyme catalytic subunit
E5R	+	Primase D5
F1L	+	Poly(A) polymerase catalytic subunit
F4L	+	DNA-directed RNA polymerase 30 kDa polypeptide
F9L	+	DNA polymerase
G11L	+	Protein F11
G12L	+	Protein F12
G15L	+	Protein F15
G1L	+	Mitochondria-associated apoptosis inhibitor, caspase-9 inhibitor, BCL2-like
G3L	+	Kelch repeat protein F3
G4L	+	Ribonucleoside-diphosphate reductase small chain
H5R	+	Putative nuclease G5

I2R	+	Ankyrin repeat protein C9L
K1R	+	Putative uncharacterized protein
L3L	+	SsDNA-binding phosphoprotein
L4L	+	Ribonucleoside-diphosphate reductase large subunit
M1L	+	Interferon antagonist K1L
M2L	+	Serine protease inhibitor-like protein SPI-3
M5L	+	Putative monoglyceride lipase f2
O1R	+	Cap-specific mRNA (nucleoside-2'-O-)-methyltransferase
O2R	+	DNA-directed RNA polymerase 22 kDa subunit
O4R	+	DNA-directed RNA polymerase 147 kDa polypeptide
P1L	+	Ankyrin repeat protein M1
P2L	+	Protein M2
R1L	+	Protein O1
A11L	-	Major core protein 4a precursor
A39R	-	Putative uncharacterized protein
A46R	-	Putative uncharacterized protein
A4L	-	Major core protein 4b
G11L	-	Protein F11
G5L	-	36 kDa major membrane protein F5
H4L	-	Glutaredoxin-2, VACV G4
J5R	-	VLTF-4 viral late transcription factor

Table S8: Host proteins degraded by the proteasome.

UniProt accession	Protein name
E7ENZ3	T-complex protein 1 subunit epsilon
1433E	Isoform SV of 14-3-3 protein epsilon
4F2	Isoform 2 of 4F2 cell-surface antigen heavy chain
A0A087WXQ8	Proteasome subunit beta type-3
ACOD	Acyl-CoA desaturase
AFF4	Isoform 2 of AF4/FMR2 family member 4
ASCC3	Activating signal cointegrator 1 complex subunit 3
ASPM	Abnormal spindle-like microcephaly-associated protein
AT2B4	Isoform ZK of Plasma membrane calcium-transporting ATPase 4
B1ALC0	Actin-related protein 2/3 complex subunit 5
B3KM87	Matrin-3
B4DK69	Aldo-keto reductase family 1 member C2
B4DLR8	NAD(P)H dehydrogenase [quinone] 1
C9J425	Fatty acid desaturase 1
C9JKI3	Caveolin
CCD50	Coiled-coil domain-containing protein 50

CTGE2	cTAGE family member 2
DAG1	Dystroglycan
DEGS1	Sphingolipid delta(4)-desaturase DES1
DUS1	Dual specificity protein phosphatase 1
E5RFT1	Zinc transporter ZIP14
E5RI99	60S ribosomal protein L30
E5RIU6	Cyclin-dependent kinase 1
E7EN77	Condensin complex subunit 1
E7ESE0	60S ribosomal protein L9
E9PF10	Nuclear pore complex protein Nup155
E9PL01	Signal peptidase complex subunit 2
E9PR16	Nuclear pore complex protein Nup160
ENOA	Alpha-enolase
F5GYT8	Methylcrotonoyl-CoA carboxylase subunit alpha, mitochondrial
F5GZ90	Denticleless protein homolog
F5H2X1	Ubiquitin carboxyl-terminal hydrolase
F5H6X6	Neutral alpha-glucosidase AB
F8VPF3	Myosin light polypeptide 6
F8W1G3	Bax inhibitor 1
F8W7C6	60S ribosomal protein L10
FADS2	Isoform 4 of Fatty acid desaturase 2
FAS	Fatty acid synthase
FDFT	Isoform 4 of Squalene synthase
GNAI3	Guanine nucleotide-binding protein G(k) subunit alpha
GNAS2	Guanine nucleotide-binding protein G(s) subunit alpha isoforms short
H0Y720	Trinucleotide repeat-containing gene 6B protein
H0Y804	Aldo-keto reductase family 1 member C1
H0Y8T0	N-alpha-acetyltransferase 11
H0Y9P0	Receptor of-activated protein C kinase 1
H0YAW4	Eukaryotic translation initiation factor 3 subunit E
H0YB22	40S ribosomal protein S14
H0YBG3	Myc proto-oncogene protein
H12	Histone H1.2
H15	Histone H1.5
H2AY	Core histone macro-H2A.2
H3BP78	Fanconi anemia group I protein
H3BQN4	Fructose-bisphosphate aldolase
H7BXK9	ATP-binding cassette sub-family B member 6, mitochondrial
HSPB1	Heat shock protein beta-1
I3L192	Basigin
IER2	Immediate early response gene 2 protein
IF2G	Eukaryotic translation initiation factor 2 subunit 3
IF4A1	Eukaryotic initiation factor 4A-I
IL32	Isoform 4 of Interleukin-32
J3QR09	Ribosomal protein L19

K2C7	Keratin, type II cytoskeletal 7
K7ENJ0	UV excision repair protein RAD23 homolog A
K7ER16	Phenylalanine--tRNA ligase alpha subunit
KPYM	Isoform M1 of Pyruvate kinase PKM
LDHB	L-lactate dehydrogenase B chain
M0R210	40S ribosomal protein S16
MOT1	Monocarboxylate transporter 1
MRP1	Isoform 8 of Multidrug resistance-associated protein 1
NDUA4	Cytochrome c oxidase subunit NDUFA4
NONO	Isoform 2 of Non-POU domain-containing octamer-binding protein
NSF1C	Isoform 4 of NSFL1 cofactor p47
PLD1	Isoform PLD1C of Phospholipase D1
PRKDC	Isoform 2 of DNA-dependent protein kinase catalytic subunit
PRP8	Pre-mRNA-processing-splicing factor 8
PSB2	Proteasome subunit beta type-2
PSB4	Proteasome subunit beta type-4
PSDE	26S proteasome non-ATPase regulatory subunit 14
PSMD7	26S proteasome non-ATPase regulatory subunit 7
Q5T8U2	60S ribosomal protein L7a
RL11	Isoform 2 of 60S ribosomal protein L11
RL15	60S ribosomal protein L15
RL3	60S ribosomal protein L3
RNH2A	Ribonuclease H2 subunit A
RS10	40S ribosomal protein S10
RS23	40S ribosomal protein S23
RS27A	Ubiquitin-40S ribosomal protein S27a
S10A4	Protein S100-A4
S19A1	Isoform 2 of Folate transporter 1
S29A1	Equilibrative nucleoside transporter 1
S43A3	Solute carrier family 43 member 3
SND1	Staphylococcal nuclease domain-containing protein 1
SUMO2	Isoform 2 of Small ubiquitin-related modifier 2
SYTC	Threonine--tRNA ligase, cytoplasmic
TCP4	Activated RNA polymerase II transcriptional coactivator p15
TCPZ	Isoform 2 of T-complex protein 1 subunit zeta
TERA	Transitional endoplasmic reticulum ATPase
TKT	Transketolase
TRI25	E3 ubiquitin/ISG15 ligase TRIM25
VIME	Vimentin
